# Uncertainty and Dimensional Calibrations

**DOI:** 10.6028/jres.102.044

**Published:** 1997

**Authors:** Ted Doiron, John Stoup

**Affiliations:** National Institute of Standards and Technology, Gaithersburg, MD 20899-0001

**Keywords:** angle standards, calibration, dimensional metrology, gage blocks, gages, optical flats, uncertainty, uncertainty budget

## Abstract

The calculation of uncertainty for a measurement is an effort to set reasonable bounds for the measurement result according to standardized rules. Since every measurement produces only an estimate of the answer, the primary requisite of an uncertainty statement is to inform the reader of how sure the writer is that the answer is in a certain range. This report explains how we have implemented these rules for dimensional calibrations of nine different types of gages: gage blocks, gage wires, ring gages, gage balls, roundness standards, optical flats indexing tables, angle blocks, and sieves.

## 1. Introduction

The calculation of uncertainty for a measurement is an effort to set reasonable bounds for the measurement result according to standardized rules. Since every measurement produces only an estimate of the answer, the primary requisite of an uncertainty statement is to inform the reader of how sure the writer is that the answer is in a certain range. Perhaps the best uncertainty statement ever written was the following from Dr. C. H. Meyers, reporting on his measurements of the heat capacity of ammonia:

“We think our reported value is good to 1 part in 10 000: we are willing to bet our own money at even odds that it is correct to 2 parts in 10 000. Furthermore, if by any chance our value is shown to be in error by more than 1 part in 1000, we are prepared to eat the apparatus and drink the ammonia.”

Unfortunately the statement did not get past the NBS Editorial Board and is only preserved anecdotally [1]. The modern form of uncertainty statement preserves the statistical nature of the estimate, but refrains from uncomfortable personal promises. This is less interesting, but perhaps for the best.

There are many “standard” methods of evaluating and combining components of uncertainty. An international effort to standardize uncertainty statements has resulted in an ISO document, “Guide to the Expression of Uncertainty in Measurement,” [2]. NIST endorses this method and has adopted it for all NIST work, including calibrations, as explained in NIST Technical Note 1297, “Guidelines for Evaluating and Expressing the Uncertainty of NIST Measurement Results” [3]. This report explains how we have implemented these rules for dimensional calibrations of nine different types of gages: gage blocks, gage wires, ring gages, gage balls, roundness standards, optical flats indexing tables, angle blocks, and sieves.

## 2. Classifying Sources of Uncertainty

Uncertainty sources are classified according to the evaluation method used. Type A uncertainties are evaluated statistically. The data used for these calculations can be from repetitive measurements of the work piece, measurements of check standards, or a combination of the two. The Engineering Metrology Group calibrations make extensive use of comparator methods and check standards, and this data is the primary source for our evaluations of the uncertainty involved in transferring the length from master gages to the customer gage. We also keep extensive records of our customers’ calibration results that can be used as auxiliary data for calibrations that do not use check standards.

Uncertainties evaluated by any other method are called Type B. For dimensional calibrations the major sources of Type B uncertainties are thermometer calibrations, thermal expansion coefficients of customer gages, deformation corrections, index of refraction corrections, and apparatus-specific sources.

For many Type B evaluations we have used a “worst case” argument of the form, “we have never seen effect X larger than Y, so we will estimate that X is represented by a rectangular distribution of half-width Y.” We then use the rules of NIST Technical Note 1297, paragraph 4.6, to get a standard uncertainty (i.e., one standard deviation estimate). It is always difficult to assess the reliability of an uncertainty analysis. When a metrologist estimates the “worst case” of a possible error component, the value is dependent on the experience, knowledge, and optimism of the estimator. It is also known that people, even experts, often do not make very reliable estimates. Unfortunately, there is little literature on how well experts estimate. Those which do exist are not encouraging [4,5].

In our calibrations we have tried to avoid using “worst case” estimates for parameters that are the largest, or near largest, sources of uncertainty. Thus if a “worst case” estimate for an uncertainty source is large, calibration histories or auxiliary experiments are used to get a more reliable and statistically valid evaluation of the uncertainty.

We begin with an explanation of how our uncertainty evaluations are made. Following this general discussion we present a number of detailed examples. The general outline of uncertainty sources which make up our generic uncertainty budget is shown in Table 1.

## 3. The Generic Uncertainty Budget

In this section we shall discuss each component of the generic uncertainty budget. While our examples will focus on NIST calibration, our discussion of uncertainty components will be broader and includes some suggestions for industrial calibration labs where the very low level of uncertainty needed for NIST calibrations is inappropriate.

### 3.1 Master Gage Calibration

Our calibrations of customer artifacts are nearly always made by comparison to master gages calibrated by interferometry. The uncertainty budgets for calibration of these master gages obviously do not have this uncertainty component. We present one example of this type of calibration, the interferometric calibration of gage blocks. Since most industry calibrations are made by comparison methods, we have focused on these methods in the hope that the discussion will be more relevant to our customers and aid in the preparation of their uncertainty budgets.

For most industry calibration labs the uncertainty associated with the master gage is the reported uncertainty from the laboratory that calibrated the master gage. If NIST is not the source of the master gage calibrations it is the responsibility of the calibration laboratory to understand the uncertainty statements reported by their calibration source and convert them, if necessary, to the form specified in the ISO Guide.

In some cases the higher echelon laboratory is accredited for the calibration by the National Voluntary Laboratory Accreditation Program (NVLAP) administered by NIST or some other equivalent accreditation agency. The uncertainty statements from these laboratories will have been approved and tested by the accreditation agency and may be used with reasonable assurance of their reliabilities.

Calibration uncertainties from non-accredited laboratories may or may not be reasonable, and some form of assessment may be needed to substantiate, or even modify, the reported uncertainty. Assessment of a laboratory’s suppliers should be fully documented.

If the master gage is calibrated in-house by intrinsic methods, the reported uncertainty should be documented like those in this report. A measurement assurance program should be maintained, including periodic measurements of check standards and interlaboratory comparisons, for any absolute measurements made by a laboratory. The uncertainty budget will not have the master gage uncertainty, but will have all of the remaining components. The first calibration discussed in Part 2, gage blocks measured by interferometry, is an example of an uncertainty budget for an absolute calibration. Further explanation of the measurement assurance procedures for NIST gage block calibrations is available [6].

### 3.2 Long Term Reproducibility

Repeatability is a measure of the variability of multiple measurements of a quantity under the same conditions over a short period of time. It is a component of uncertainty, but in many cases a fairly small component. It might be possible to list the changes in conditions which could cause measurement variation, such as operator variation, thermal history of the artifact, electronic noise in the detector, but to assign accurate quantitative estimates to these causes is difficult. We will not discuss repeatability in this paper.

What we would really like for our uncertainty budget is a measure of the variability of the measurement caused by all of the changes in the measurement conditions commonly found in our laboratory. The term used for the measure of this larger variability caused by the changing conditions in our calibration system is reproducibility.

The best method to determine reproducibility is to compare repeated measurements over time of the same artifact from either customer measurement histories or check standard data. For each dimensional calibration we use one or both methods to evaluate our long term reproducibility.

We determine the reproducibility of absolute calibrations, such as the dimensions of our master artifacts, by analyzing the measurement history of each artifact. For example, a gage block is not used as a master until it is measured 10 times over a period of 3 years. This ensures that the block measurement history includes variations from different operators, instruments, environmental conditions, and thermometer and barometer calibrations. The historical data then reflects these sources in a realistic and statistically valid way. The historical data are fit to a straight line and the deviations from the best fit line are used to calculate the standard deviation.

The use of historical data (master gage, check standard, or customer gage) to represent the variability from a particular source is a recurrent theme in the example presented in this paper. In each case there are two conditions which need to be met:

First, the measurement history must sample the sources of variation in a realistic way. This is a particular concern for check standard data. The check standards must be treated as much like a customer gage as possible.

Second, the measurement history must contain enough changes in the source of variability to give a statistically valid estimate of its effect. For example, the standard platinum resistance thermometer (SPRT) and barometers are recalibrated on a yearly basis, and thus the measurement history must span a number of years to sample the variability caused by these sensor calibrations.

For most comparison measurements we use two NIST artifacts, one as the master reference and the other as a check standard. The customer’s gage and both NIST gages are measured two to six times (depending on the calibration) and the lengths of the customer block and check standard are derived from a least-squares fit of the measurement data to an analytical model of the measurement scheme [7]. The computer records the measured difference in length between the two NIST gages for every calibration. At the end of each year the data from all of the measurement stations are sorted by size into a single history file. For each size, the data from the last few years is collected from the history files. A least-squares method is used to find the best-fit line for the data, and the deviations from this line are used to calculate the estimated standard deviation, s [8,9]. This s is used as the estimate of the reproducibility of the comparison process.

If one or both of the master artifacts are not stable, the best fit line will have a non-zero slope. We replace the block if the slope is more than a few nanometers per year.

There are some calibrations for which it is impractical to have check standards, either for cost reasons or because of the nature of the calibration. For example, we measure so few ring standards of any one size that we do not have many master rings. A new gage block stack is prepared as a master gage for each ring calibration. We do, however, have several customers who send the same rings for calibration regularly, and these data can be used to calculate the reproducibility of our measurement process.

### 3.3 Thermal Expansion

All dimensions reported by NIST are the dimensions of the artifact at 20 °C. Since the gage being measured may not be exactly at 20 °C, and all artifacts change dimension with temperature change, there is some uncertainty in the length due to the uncertainty in temperature. We correct our measurements at temperature *t* using the following equation:

ΔL=α(20°C−t)L (1)

where *L* is the artifact length at celsius temperature *t*, Δ*L* is the length correction, *α* is the coefficient of thermal expansion (CTE), and *t* is the artifact temperature.

This equation leads to two sources of uncertainty in the correction Δ*L*: one from the temperature standard uncertainty, *u*(*t*), and the other from the CTE standard uncertainty, *u*(*α*):

$$U^{2}\left(\delta L\right)={\left[\alpha L\cdot u\left(t\right)\right]}^{2}+{\left[L\left(20\;{}^{\circ}C-t\right)u\left(\alpha \right)\right]}^{2}.$$ (2)

The first term represents the uncertainty due to the thermometer reading and calibration. We use a number of different types of thermometers, depending on the required measurement accuracy. Note that for comparison measurements, if both gages are made of the same material (and thus the same nominal CTE), the correction is the same for both gages, no matter what the temperature uncertainty. For gages of different materials, the correction and uncertainty in the correction is proportional to the difference between the CTEs of the two materials.

The second term represents the uncertainty due to our limited knowledge of the real CTE for the gage. This source of uncertainty can be made arbitrarily small by making the measurements suitably close to 20 °C.

Most comparison measurements rely on one thermometer near or attached to one of the gages. For this case there is another source of uncertainty, the temperature difference between the two gages. Thus, there are three major sources of uncertainty due to temperature.

- The thermometer used to measure the temperature of the gage has some uncertainty.
- If the measurement is not made at exactly 20 °C, a thermal expansion correction must be made using an assumed thermal expansion coefficient. The uncertainty in this coefficient is a source of uncertainty.
- In comparison calibrations there can be a temperature difference between the master gage and the test gage.

#### 3.3.1 Thermometer Calibration

We used two types of thermometers. For the highest accuracy we used thermocouples referenced to a calibrated long stem SPRT calibrated at NIST with an uncertainty (3 standard deviation estimate) equivalent to 0.001 °C. We own four of these systems and have tested them against each other in pairs and chains of three. The systems agree to better than 0.002 °C. Assuming a rectangular distribution with a half-width of 0.002 °C, we get a standard uncertainty of $0.002\;{}^{\circ}C/\sqrt{3}=0/0012\;{}^{\circ}C$. Thus *u*(*t*) = 0.0012 °C for SPRT/thermocouple systems.

For less critical applications we use thermistor based digital thermometers calibrated against the primary platinum resistors or a transfer platinum resistor. These thermistors have a least significant digit of 0.01 °C. Our calibration history shows that the thermistors drift slowly with time, but the calibration is never in error by more than ±0.02 °C. Therefore we assume a rectangular distribution of half-width of 0.02 °C, and obtain $u\left(t\right)=0.02\;{}^{\circ}C/\sqrt{3}=0.012\;{}^{\circ}C$ for the thermistor systems.

In practice, however, things are more complicated. In the cases where the thermistor is mounted on the gage there are still gradients within the gage. For absolute measurements, such as gage block interferometry, we use one thermometer for each 100 mm of gage length. The average of these readings is taken as the gage temperature.

#### 3.3.2 Coefficient of Thermal Expansion (CTE)

The uncertainty associated with the coefficient of thermal expansion depends on our knowledge of the individual artifact. Direct measurements of CTEs of the NIST steel master gage blocks make this source of uncertainty very small. This is not true for other NIST master artifacts and nearly all customer artifacts. The limits allowable in the ANSI [19] gage block standard are ±1×10^−6^/°C. Until recently we have assumed that this was an adequate estimate of the uncertainty in the CTE. The variation in CTEs for steel blocks, for our earlier measurements, is dependent on the length of the block. The CTE of hardened gage block steel is about 12×10^−6^/°C and unhardened steel 10.5×10^−6^/°C. Since only the ends of long gage blocks are hardened, at some length the middle of the block is unhardened steel. This mixture of hardened and unhardened steel makes different parts of the block have different coefficients, so that the overall coefficient becomes length dependent. Our previous studies found that blocks up to 100 mm long were completely hardened steel with CTEs near 12×10^−6^/°C. The CTE then became lower, proportional to the length over 100 mm, until at 500 mm the coefficients were near 10.5×10^−6^/°C. All blocks we had measured in the past followed this pattern.

Recently we have calibrated a long block set which had, for the 20 in block, a CTE of 12.6×10^−6^/°C. This experience has caused us to expand our worst case estimate of the variation in CTE from ±1×10^−6^/°C to ±2×10^−6^/°C, at least for long steel blocks for which we have no thermal expansion data. Taking 2×10^−6^/°C as the half-width of a rectangular distribution yields a standard uncertainty of $u\left(\alpha \right)=\left(2\times 10^{-6}/{}^{\circ}C\right)/\sqrt{3}=1.2\times 10^{-6}\;{}^{\circ}C$ for long hardened steel blocks.

For other materials such as chrome carbide, ceramic, etc., there are no standards and the variability from the manufacturers nominal coefficient is unknown. Handbook values for these materials vary by as much as 1×10^−6^/°C. Using this as the half-width of a rectangular distribution yields a standard uncertainty of $u\left(\alpha \right)=\left(2\times 10^{-6}/{}^{\circ}C\right)/\sqrt{3}=0.6\times 10^{-6}\;{}^{\circ}C$ for materials other than steel.

#### 3.3.3 Thermal Gradients

For small gages the thermistor is mounted near the measured gage but on a different (similar) gage. For example, in gage block comparison measurements the thermometer is on a separate block placed at the rear of the measurement anvil. There can be gradients between the thermistor and the measured gage, and differences in temperature between the master and customer gages. Estimating these effects is difficult, but gradients of up to 0.03 °C have been measured between master and test artifacts on nearly all of our measuring equipment. Assuming a rectangular distribution with a half-width of 0.03 °C we obtain a standard uncertainty of $u\left(\Delta t\right)=0.03\;{}^{\circ}C/\sqrt{3}=0.017\;{}^{\circ}C$. We will use this value except for specific cases studied experimentally.

### 3.4 Mechanical Deformation

All mechanical measurements involve contact of surfaces and all surfaces in contact are deformed. In some cases the deformation is unwanted, in gage block comparisons for example, and we apply a correction to get the undeformed length. In other cases, particularly thread wires, the deformation under specified conditions is part of the length definition and corrections may be needed to include the proper deformation in the final result.

The geometries of deformations occurring in our calibrations include:

1. Sphere in contact with a plane (for example, gage blocks)
2. Sphere in contact with an internal cylinder (for example, plain ring gages)
3. Cylinders with axes crossed at 90° (for example, cylinders and wires)
4. Cylinder in contact with a plane (for example, cylinders and wires).

In comparison measurements, if both the master and customer gages are made of the same material, the deformation is the same for both gages and there is no need for deformation corrections. We now use two sets of master gage blocks for this reason. Two sets, one of steel and one of chrome carbide, allow us to measure 95 % of our customer blocks without corrections for deformation.

At the other extreme, thread wires have very large applied deformation corrections, up to 1 μm (40 μin). Some of our master wires are measured according to standard ANSI/ASME B1 [10] conditions, but many are not. Those measured between plane contacts or between plane and cylinder contacts not consistent with the B1 conditions require large corrections. When the master wire diameter is given at B1 conditions (as is done at NIST), calibrations using comparison methods do not need further deformation corrections.

The equations from “Elastic Compression of Spheres and Cylinders at Point and Line Contact,” by M. J. Puttock and E. G. Thwaite, [11] are used for all deformation corrections. These formulas require only the elastic modulus and Poisson’s ratio for each material, and provide deformation corrections for contacts of planes, spheres, and cylinders in any combination.

The accuracy of the deformation corrections is assessed in two ways. First, we have compared calculations from Puttock and Thwaite with other published calculations, particularly with NBS Technical Note 962, “Contact Deformation in Gage Block Comparisons” [12] and NBSIR 73-243, “On the Compression of a Cylinder in Contact With a Plane Surface” [13]. In all of the cases considered the values from the different works were within 0.010 μm (0.4 μin). Most of this difference is traceable to different assumptions about the elastic modulus of “steel” made in the different calculations.

The second method to assess the correction accuracy is to make experimental tests of the formulas. A number of tests have been performed with a micrometer developed to measure wires. One micrometer anvil is flat and the other a cylinder. This allows wire measurements in a configuration much like the defined conditions for thread wire diameter given in ANSI/ASME B1 Screw Thread Standard. The force exerted by the micrometer on the wire is variable, from less than 1 N to 10 N. The force gage, checked by loading with small calibrated masses, has never been incorrect by more than a few per cent. This level of error in force measurement is negligible.

The diameters measured at various forces were corrected using calculated deformations from Puttock and Thwaite. The deviations from a constant diameter are well within the measurement scatter, implying that the corrections from the formula are smaller than the measurement variability. This is consistent with the accuracy estimates obtained from comparisons reported in the literature.

For our estimate we assume that the calculated corrections may be modeled by a rectangular distribution with a half-width of 0.010 μm. The standard uncertainty is then $u\left(\text{def}\right)=0.010\;\mu m/\sqrt{3}=0.006\;\mu m$.

Long end standards can be measured either vertically or horizontally. In the vertical orientation the standard will be slightly shorter, compressed under its own weight. The formula for the compression of a vertical column of constant cross-section is

$$\Delta \left(L\right)=\frac{\rho gL^{2}}{2E}$$ (3)

where *L* is the height of the column, *E* is the external pressure, *ρ* is the density of the column, and *g* is the acceleration of gravity.

This correction is less than 25 nm for end standards under 500 mm. The relative uncertainties of the density and elastic modulus of steel are only a few percent; the uncertainty in this correction is therefore negligible.

### 3.5 Scale Calibration

Since the meter is defined in terms of the speed of light, and the practical access to that definition is through comparisons with the wavelength of light, all dimensional measurements ultimately are traceable to an interferometric measurement [14]. We use three types of scales for our measurements: electronic or mechanical transducers, static interferometry, and displacement interferometry.

The electronic or mechanical transducers generally have a very short range and are calibrated using artifacts calibrated by interferometry. The uncertainty of the sensor calibration depends on the uncertainty in the artifacts and the reproducibility of the sensor system. Several artifacts are used to provide calibration points throughout the sensor range and a least-squares fit is used to determine linear calibration coefficients.

The main forms of interferometric calibration are static and dynamic interferometry. Distance is measured by reading static fringe fractions in an interferometer (e.g., gage blocks). Displacement is measured by analyzing the change in the fringes (fringe counting displacement interferometer). The major sources of uncertainty—those affecting the actual wavelength—are the same for both methods. The uncertainties related to actual data readings and instrument geometry effects, however, depend strongly on the method and instruments used.

The wavelength of light depends on the frequency, which is generally very stable for light sources used for metrology, and the index of refraction of the medium the light is traveling through. The wavelength, at standard conditions, is known with a relative standard uncertainty of 1×10^−7^ or smaller for most commonly used atomic light sources (helium, cadmium, sodium, krypton). Several types of lasers have even smaller standard uncertainties—1×10^−10^ for iodine stabilized HeNe lasers, for example. For actual measurements we use secondary stabilized HeNe lasers with relative standard uncertainties of less than 1×10^−8^ obtained by comparison to a primary iodine stabilized laser. Thus the uncertainty associated with the frequency (or vacuum wavelength) is negligible.

For measurements made in air, however, our concern is the uncertainty of the wavelength. If the index of refraction is measured directly by a refractometer, the uncertainty is obtained from an uncertainty analysis of the instrument. If not, we need to know the index of refraction of the air, which depends on the temperature, pressure, and the molecular content. The effect of each of these variables is known and an equation to make corrections has evolved over the last 100 years. The current equation, the Edlén equation, uses the temperature, pressure, humidity and CO_2_ content of the air to calculate the index of refraction needed to make wavelength corrections [15]. Table 2 shows the approximate sensitivities of this equation to changes in the environment.

Other gases affect the index of refraction in significant ways. Highly polarizable gases such as Freons and organic solvents can have measurable effects at surprisingly low concentrations [16]. We avoid using solvents in any area where interferometric measurements are made. This includes measuring machines, such as micrometers and coordinate measuring machines, which use displacement interferometers as scales.

Table 2 can be used to estimate the uncertainty in the measurement for each of these sources. For example, if the air temperature in an interferometric measurement has a standard uncertainty of 0.1 °C, the relative standard uncertainty in the wavelength is 0.1×10^−6^ μm/m. Note that the wavelength is very sensitive to air pressure: 1.2 kPa to 4 kPa changes during a day, corresponding to relative changes in wavelength of 3×10^−6^ to 10^−5^ are common. For high accuracy measurements the air pressure must be monitored almost continuously.

### 3.6 Instrument Geometry

Each instrument has a characteristic motion or geometry that, if not perfect, will lead to errors. The specific uncertainty depends on the instrument, but the sources fall into a few broad categories: reference surface geometry, alignment, and motion errors.

Reference surface geometry includes the flatness and parallelism of the anvils of micrometers used in ball and cylinder measurements, the roundness of the contacts in gage block and ring comparators, and the sphericity of the probe balls on coordinate measuring machines. It also includes the flatness of reference flats used in many interferometric measurements.

The alignment error is the angle difference and offset of the measurement scale from the actual measurement line. Examples are the alignment of the two opposing heads of the gage block comparator, the laser or LVDT alignment with the motion axis of micrometers, and the illumination angle of interferometers.

An instrument such as a micrometer or coordinate measuring machine has a moving probe, and motion in any single direction has six degrees of freedom and thus six different error motions. The scale error is the error in the motion direction. The straightness errors are the motions perpendicular to the motion direction. The angular error motions are rotations about the axis of motion (roll) and directions perpendicular to the axis of motion (pitch and yaw). If the scale is not exactly along the measurement axis the angle errors produce measurement errors called Abbe errors.

**Fig. 1:**
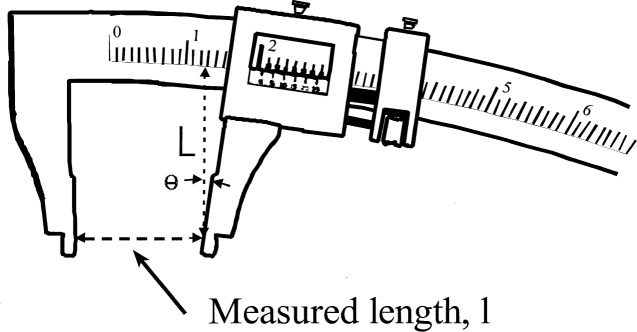
The Abbe error is the product of the perpendicular distance of the scale from the measuring point, *L*, times the sine of the pitch angle error, *Θ*, error = *L* sin*Θ*.

In Fig. 1 the measuring scale is not straight, giving a pitch error. The size of the error depends on the distance *L* of the measured point from the scale and the angular error 1. For many instruments this Abbe offset *L* is not near zero and significant errors can be made.

The geometry of gage block interferometers includes two corrections that contribute to the measurement uncertainty. If the light source is larger than 1 mm in any direction (a slit for example) a correction must be made. If the light path is not orthogonal to the surface of the gage there is also a correction related to cosine errors called obliquity correction. Comparison of results between instruments with different geometries is an adequate check on the corrections supplied by the manufacturer.

### 3.7 Artifact Effects

The last major sources of uncertainty are the properties of the customer artifact. The most important of these are thermal and geometric. The thermal expansion of customer artifacts was discussed earlier (Sec. 3.3).

Perhaps the most difficult source of uncertainty to evaluate is the effect of the test gage geometry on the calibration. We do not have time, and it is not economically feasible, to check the detailed geometry of every artifact we calibrate. Yet we know of many artifact geometry flaws that can seriously affect a calibration.

We test the diameter of gage balls by repeated comparisons with a master ball. Generally, the ball is measured in a random orientation each time. If the ball is not perfectly round the comparison measurements will have an added source of variability as we sample different diameters of the ball. If the master ball is not round it will also add to the variability. The check standard measurement samples this error in each measurement.

Gage wires can have significant taper, and if we measure the wire at one point and the customer uses it at a different point our reported diameter will be wrong for the customer’s measurement. It is difficult to estimate how much placement error a competent user of the wire would make, and thus difficult to include such effects in the uncertainty budget. We have made assumptions on the basis of how well we center the wires by eye on our equipment.

We calibrate nearly all customer gage blocks by mechanical comparison to our master gage blocks. The length of a master gage block is determined by interferometric measurements. The definition of length for gage blocks includes the wringing layer between the block and the platen. When we make a mechanical comparison between our master block and a test block we are, in effect, assigning our wringing layer to the test block. In the last 100 years there have been numerous studies of the wringing layer that have shown that the thickness of the layer depends on the block and platen flatness, the surface finish, the type and amount of fluid between the surfaces, and even the time the block has been wrung down. Unfortunately, there is still no way to predict the wringing layer thickness from auxiliary measurements. Later we will discuss how we have analyzed some of our master blocks to obtain a quantitative estimate of the variability.

For interferometric measurements, such as gage blocks, which involve light reflecting from a surface, we must make a correction for the phase shift that occurs. There are several methods to measure this phase shift, all of which are time consuming. Our studies show that the phase shift at a surface is reasonably consistent for any one manufacturer, material, and lapping process, so that we can assign a “family” phase shift value to each type and source of gage blocks. The variability in each family is assumed small. The phase shift for good quality gage block surfaces generally corresponds to a length offset of between zero (quartz and glass) and 60 nm (steel), and depends on both the materials and the surface finish. Our standard uncertainty, from numerous studies, is estimated to be less than 10 nm.

Since these effects depend on the type of artifact, we will postpone further discussion until we examine each calibration.

### 3.8 Calculation of Uncertainty

In calculating the uncertainty according to the ISO Guide [2] and NIST Technical Note 1297 [3], individual standard uncertainty components are squared and added together. The square root of this sum is the combined standard uncertainty. This standard uncertainty is then multiplied by a coverage factor *k*. At NIST this coverage factor is chosen to be 2, representing a confidence level of approximately 95 %.

When length-dependent uncertainties of the form *a*+*bL* are squared and then added, the square root is not of the form *a*+*bL*. For example, in one calibration there are a number of length-dependent and length-independent terms:

$$\begin{array}{l} u_{1}=0.12\;\mu m\\ u_{2}=0.07\;\mu m+0.03\times 10^{-6}L\\ u_{3}=0.08\times 10^{-6}L\\ u_{4}=0.23\times 10^{-6}L \end{array}$$

**Fig. 2:**
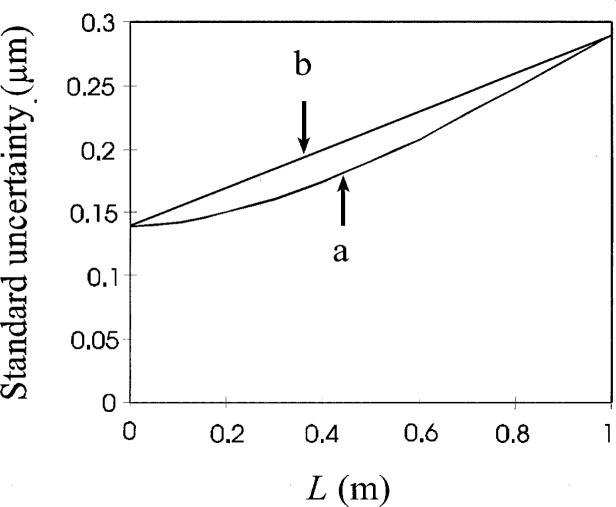
The standard uncertainty of a gage block as a function of length (a) and the linear approximation (b).

If we square each of these terms, sum them, and take the square root we get the lower curve in Fig. 2.

Note that it is not a straight line. For convenience we would like to preserve the form *a*+*bL* in our total uncertainty, we must choose a line to approximate this curve. In the discussions to follow we chose a length range and approximate the uncertainty by taking the two end points on the calculated uncertainty curve and use the straight line containing those points as the uncertainty. In this example, the uncertainty for the range 0 to 1 length units would be the line *f = a*+*bL* containing the points (0, 0.14 μm) and (1, 0.28 μm).

Using a coverage factor *k* = 2 we get an expanded uncertainty *U* of *U* = 0.28 μm+0.28×10^−6^ *L* for *L* between 0 and 1. Most cases do not generate such a large curvature and the overestimate of the uncertainty in the mid-range is negligible.

### 3.9 Uncertainty Budgets for Individual Calibrations

In the remaining sections we discuss the uncertainty budgets of calibrations performed by the NIST Engineering Metrology Group. For each calibration we list and discuss the sources of uncertainty using the generic uncertainty budget as a guide. At the end of each discussion is a formal uncertainty budget with typical values and calculated total uncertainty.

Note that we use a number of different calibration methods for some types of artifacts. The method chosen depends on the requested accuracy, availability of master standards, or equipment. We have chosen one method for each calibration listed below.

Further, many calibrations have uncertainties that are very sensitive to the size and condition of the artifact. The uncertainties shown are for “typical” customer calibrations. The uncertainty for any individual calibration may differ considerably from the results in this work because of the quality of the customer gage or changes in our procedures.

The calibrations discussed are:

Gage blocks (interferometry)
Gage blocks (mechanical comparison)
Gage wires (thread and gear wires) and cylinders (plug gages)
Ring gages (diameter)
Gage balls (diameter)
Roundness standards (balls, rings, etc.)
Optical flats Indexing tables
Angle blocks
Sieves

The calibration of line scales is discussed in a separate document [17].

## 4. Gage Blocks (Interferometry)

The NIST master gage blocks are calibrated by interferometry using a calibrated HeNe laser as the light source [18]. The laser is calibrated against an iodine-stabilized HeNe laser. The frequency of stabilized lasers has been measured by a number of researchers and the current consensus values of different stabilized frequencies are published by the International Bureau of Weights and Measures [12]. Our secondary stabilized lasers are calibrated against the iodine-stabilized laser using a number of different frequencies.

### 4.1 Master Gage Calibration

This calibration does not use master reference gages.

### 4.2 Long Term Reproducibility

The NIST master gage blocks are not used until they have been measured at least 10 times over a 3 year span. This is the minimum number of wrings we think will give a reasonable estimate of the reproducibility and stability of the block. Nearly all of the current master blocks have considerably more data than this minimum, with some steel blocks being measured more than 50 times over the last 40 years. These data provide an excellent estimate of reproducibility. In the long term, we have performed calibrations with many different technicians, multiple calibrations of environmental sensors, different light sources, and even different interferometers.

**Fig. 3:**
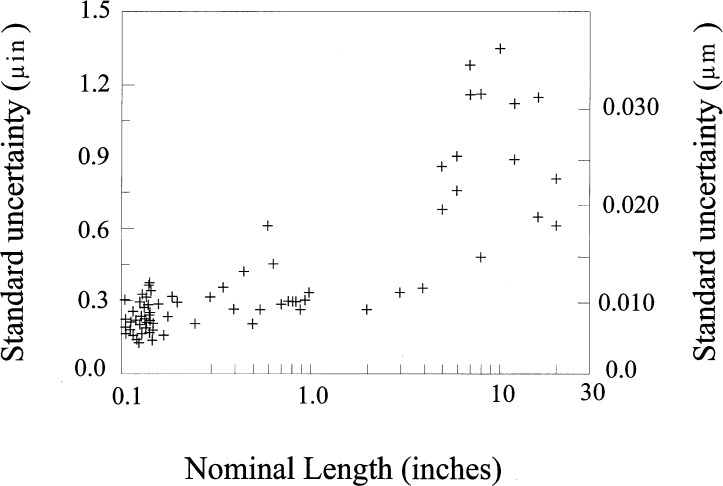
Standard deviations for interferometric calibration of NIST master gage blocks of different length as obtained over a period of 25 years.

As expected, the reproducibility is strongly length dependent, the major variability being caused by thermal properties of the blocks and measurement apparatus. The data do not, however, fall on a smooth line. The standard deviation data from our calibration history is shown in Fig. 3.

There are some blocks, particularly long blocks, which seem to have more or less variability than the trend would predict. These exceptions are usually caused by poor parallelism, flatness or surface finish of the blocks. Ignoring these exceptions the standard deviation for each length follows the approximate formula:

$$u\left(\text{rep}\right)=0.009\;\mu m+0.08\times 10^{-6}L\;\left(\text{NIST}\;\text{Masters}\right)$$ (4)

For interferometry on customer blocks the reproducibility is worse because there are fewer measurements. The numbers above represent the uncertainty of the mean of 10 to 50 wrings of our master blocks. Customer calibrations are limited to 3 wrings because of time and financial constraints. The standard deviation of the mean of *n* measurements is the standard deviation of the *n* measurements divided by the square root of *n*. We can relate the standard deviation of the mean of 3 wrings to the standard deviations from our master block history through the square root of the ratio of customer rings (3) to master block measurements (10 to 50). We will use 20 as the average number of wrings for NIST master blocks. The uncertainty of 3 wrings is then approximately 2.5 times that for the NIST master blocks. The standard uncertainty for 3 wrings is

$$u\left(\text{rep}\right)=0.022\;\mu m+0.20\times 10^{-6}L\;\left(3\;\text{wrings}\right).$$ (5)

### 4.3 Thermal Expansion

#### 4.3.1 Thermometer Calibration

The thermometers used for the calibrations have been changed over the years and their history samples multiple calibrations of each thermometer. Thus, the master block historical data already samples the variability from the thermometer calibration.

Thermistor thermometers are used for the calibration of customer blocks up to 100 mm in length. As discussed earlier [(see eq. 2)] we will take the uncertainty of the thermistor thermometers to be 0.01 °C. For longer blocks, a more accurate system consisting of a platinum SPRT (Standard Platinum Resistance Thermometer) as a reference and thermocouples is used.

#### 4.3.2 Coefficient of Thermal Expansion (CTE)

The CTE of each of our blocks over 25 mm in length has been measured, leaving a very small standard uncertainty estimated to be 0.05×10^−6^/°C. Since our measurements are always within ±0.1 °C of 20 °C, the uncertainty in length is taken to be 0.005×10^−6^ *L*.

#### 4.3.3 Thermal Gradients

The long block temperature is measured every 100 mm, reducing the effects of thermal gradients to a negligible level.

The gradients between the thermometer and test blocks in the short block interferometer (up to 100 mm) are small because the entire measurement space is in a metal enclosure. The gradients between the thermometer in the center of the platen and any block are less than 0.005 °C. Assuming a rectangular distribution with a half-width 0.005 °C, we obtain a standard uncertainty of 0.003 °C in temperature. For steel gage blocks (CTE = 11.5 μm/(m · °C)), the standard uncertainty in length is 0.003×10^−6^ *L*. For other materials the uncertainty is less.

### 4.4 Elastic Deformation

We measure blocks oriented vertically, as specified in the ANSI/ASME B89.1.9 Gage Block Standard [19]. For customers who need the length of long blocks in the horizontal orientation, a correction factor is used. This correction for self loading is proportional to the square of the length, and is very small compared to other effects. For 500 mm blocks the correction is only about 25 nm, and the uncertainty depends on the uncertainty in the elastic modulus of the gage block material. Nearly all long blocks are made of steel, and the variations in elastic modulus for gage block steels is only a few percent. The standard uncertainty in the correction is estimated to be less than 2 nm, a negligible addition to the uncertainty budget.

### 4.5 Scale Calibration

The laser is calibrated against a well characterized iodine-stabilized laser. We estimate the relative standard uncertainty in the frequency from this calibration to be less than 10^−8^, which is negligible for gage block calibrations.

The Edlén equation for the index of refraction of air, *n*, has a relative standard uncertainty of 3×10^−8^.

Customer calibrations are made under a single environmental sensor calibration cycle and the uncertainty from these sources must be estimated. We check our pressure sensors against a barometric pressure standard maintained by the NIST Pressure Group. Multiple comparisons lead us to estimate the standard uncertainty of our pressure gages is 8 Pa. The air temperature measurement has a standard uncertainty of about 0.015 °C, as discussed previously. By comparing several hygrometers we estimate that the standard uncertainty of the relative humidity is about 3 %.

The gage block historical data contains measurements made with a number of sources including elemental discharge lamps (cadmium, helium, krypton) and several calibrated lasers. The historical data, therefore, contains an adequate sampling of the light source frequency uncertainty.

### 4.6 Instrument Geometry

The obliquity and slit corrections provided by the manufacturers are used for all of our interferometers. We have tested these corrections by measuring the same blocks in all of the interferometers and have found no measurable discrepancies. Measuring blocks in interferometers of different geometries could also be used to find the corrections. For example, our Koesters type interferometer has no obliquity correction when properly aligned, and the slit is accessible for measurement. Thus, the correction can be calculated. The Hilger interferometer slits cannot be measured except by disassembly, but the corrections can be found by comparison of measurements with the Koesters interferometer.

The only geometry errors, other than those discussed above, are due to the platen flatness. Each platen is examined and is not used unless it is flat to 50 nm over the entire 150 mm diameter. Since the gage block measurement is made over less than 25 mm of the surface, the local flatness is quite good. In addition, the measurement history of the master blocks has data from many platens and multiple positions on each platen, so the variability from the platen flatness is sampled in the data.

### 4.7 Artifact Geometry

The phase change that light undergoes on reflection depends on the surface finish and the electromagnetic properties of the block material. We assume that every block from a single manufacturer of the same material has the same surface finish and material, and therefore gives rise to the same phase change. We have restricted our master blocks to a few manufacturers and materials to reduce the work needed to characterize the phase change. Samples of each material and manufacturer are measured by the slave block method [4], and these results are used for all blocks of similar material and the same manufacturer.

**Fig. 4:**
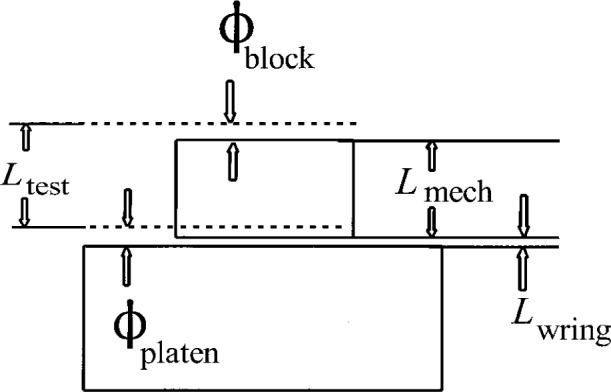
Diagram showing the phase shift *ϕ* on reflection makes the light appear to have reflected from a surface slightly above the physical metal surface.

**Fig. 5:**
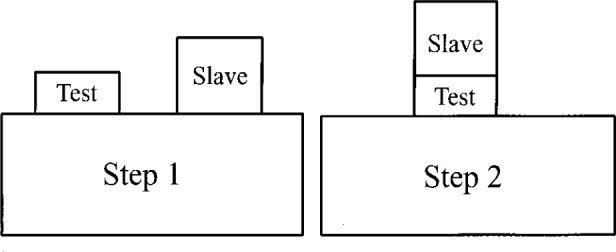
Schematic depiction of the measurements for determining the phase shift difference between a block and platen by the slave block method.

In the slave block method, an auxiliary block, called the slave block, is used to help find the phase shift difference between a block and a platen. The method consists of two steps, shown schematically in Figs. 4 and 5.

The interferometric length *L*_test_ includes the mechanical length, the wringing film thickness, and the phase change at each surface.

#### Step 1

The test and slave blocks are wrung down to the same platen and measured independently. The two lengths measured consist of the mechanical length of the block, the wringing film, and the phase changes at the top of the block and platen, as in Fig. 4.

The general formula for the measured length of a wrung block is:

$$L_{\text{test}}=L_{\text{mechanical}}+L_{\text{wring}}+L_{\text{platen}\;\text{phase}}-L_{\text{block}\;\text{phase}}.$$ (6)

For the test and slave blocks the formulas are

$$L_{\text{test}}=L_{t}+L_{t,w}+\left(\phi_{\text{platen}}-\phi_{\text{test}}\right)$$ (7)

$$L_{\text{slave}}=L_{s}+L_{s,w}+\left(\phi_{\text{platen}}-\phi_{\text{slave}}\right)$$ (8)

where *L*_t_, *L*_t,w_, *L*_s_, and *L*_s,w_ are defined in Fig. 4.

#### Step 2

Either the slave block or both blocks are taken off the platen, cleaned, and rewrung as a stack on the platen. The length of the stack measured is:

$$L_{\text{test}+\text{slave}}=L_{t}+L_{s}+L_{t,w}+L_{s,w}+\left(\phi_{\text{platen}}-\phi_{\text{slave}}\right).$$ (9)

If this result is subtracted from the sum of the two previous measurements, we find that

$$L_{\text{test}+\text{slave}}-L_{\text{test}}-L_{\text{slave}}=\left(\phi_{\text{test}}-\phi_{\text{platen}}\right).$$ (10)

The weakness of this method is the uncertainty of the measurements. The standard uncertainty of one measurement of a wrung gage block is about 0.030 μm (from the long term reproducibility of our master block calibrations). Since the phase measurement depends on three measurements, the phase measurement has a standard uncertainty of about $\sqrt{3}$ times the uncertainty of one measurement, or about 0.040 μm. Since the phase difference between block and platen is generally corresponds to a length of about 0.020 μm, the uncertainty is larger than the effect. To reduce the uncertainty, a large number of measurements must be made, generally around 50. This is, of course, very time consuming.

For our master blocks, using the average number of slave block measurements gives an estimate of 0.006 μm for the standard uncertainty due to the phase correction.

We restrict our calibration service to small (8 to 10 block) audit sets for customers who do interferometry. These audit sets are used as checks on the customer measurement process, and to assure that the uncertainty is low we restrict the blocks to those from manufacturers for which we have adequate phase-correction data. The uncertainty is, therefore, the same as for our own master blocks. On the rare occasions that we measure blocks of unknown phase, the uncertainty is very dependent on the procedure used, and is outside the scope of this paper.

If the gage block is not flat and parallel, the fringes will be slightly curved and the position on the block where the fringe fraction is measured becomes important. For our measurements we attempt to read the fringe fraction as close to the gage point as possible. However, using just the eye, this is probably uncertain to 1 mm to 2 mm. Since most blocks we measure are flat and parallel to 0.050 μm over the entire surface, the error is small. If the block is 9 mm wide and the flatness/parallelism is 0.050 μm then a 1 mm error in the gage point produces a length error of about 0.005 μm. For customer blocks this is reduced somewhat because three measurements are made, but since the readings are made by the same person operator bias is possible. We use a standard uncertainty of 0.003 μm to account for this possibility. Our master blocks are measured over many years by different technicians and the variability from operator effects are sampled in the historical data.

### 4.8 Summary

Tables 3 and 4 show the uncertainty budgets for interferometric calibration of our master reference blocks and customer submitted blocks. Using a coverage factor of *k* = 2 we obtain the expanded uncertainty *U* of our interferometer gage block calibrations for our master gage blocks as *U* = 0.022 μm+0.16×10^−6^ *L*.

The uncertainty budget for customer gage block calibrations (three wrings) is only slightly different. The reproducibility uncertainty is larger because of fewer measurements and because the thermal expansion coefficient has not been measured on customer blocks. Using a coverage factor of k=2 we obtain an expanded uncertainty *U* for customer calibrations (three wrings) of *U* = 0.05 μm+0.4×10^−6^ *L*.

## 5. Gage Blocks (Mechanical Comparison)

Most customer calibrations are made by mechanical comparison to master gage blocks calibrated on a regular basis by interferometry. The comparison process compares each gage block with two NIST master blocks of the same nominal size [20]. We have one steel and one chrome carbide master block for each standard size. The customer block length is derived from the known length of the NIST master made of the same material to avoid problems associated with deformation corrections. Deformation corrections are needed for tungsten carbide blocks and we assign higher uncertainties than those described below.

In the discussion below we group gage blocks into three groups, each with slightly different uncertainty statements. Sizes over 100 mm are measured on different instruments than those 100 mm or less, and have different measurement procedures. Thus they form a distinct process and are handled separately. Blocks under 1 mm are measured on the same equipment as those between 1 mm and 100 mm, but the blocks have different characteristics and are considered here as a separate process. The major difference is that thin blocks are generally not very flat, and this leads to an extra uncertainty component. They are also so thin that length-dependent sources of uncertainty are negligible.

### 5.1 Master Gage Calibration

From the previous analysis (see Sec. 4.8) the standard uncertainty u of the length of the NIST master blocks is *u* = 0.011 μm+0.08×10^−6^ *L*. Of course, some blocks have a longer measurement history than others, but for this discussion we use the average. We use the actual value for each master block to calculate the uncertainty reported for the customer block. Thus, numbers generated in this discussion only approximate those in an actual report.

### 5.2 Long Term Reproducibility

We use two NIST master gage blocks in every calibration, one steel and the other chrome carbide. When the customer block is steel or ceramic, the steel block length is the master (restraint in the data analysis). When the customer block is chrome or tungsten carbide, the chrome carbide block is the master. The difference between the two NIST blocks is a control parameter (check standard).

The check standard data are used to estimate the long term reproducibility of the comparison process. The two NIST blocks are of different materials so the measurements have some variability due to contact force variations (deformation) and temperature variations (differential thermal expansion). Customer calibrations, which compare like materials, are less susceptible to these sources of variability. Thus, using the check standard data could produce an overestimate of the reproducibility. We do have some size ranges where both of the NIST master blocks are steel, and the variability in these calibrations has been compared to the variability among similar sizes where we have masters of different material. We have found no significant difference, and thus consider our use of the check standard data as a valid estimate of the long term reproducibility of the system.

The standard uncertainty derived from our control data is, as expected, a smooth curve that rises slowly with the length of the blocks. For mechanical comparisons we pool the control data for similar sizes to obtain the long term reproducibility. We justify this grouping by examining the sources of uncertainty. The interferometry data are not grouped because the surface finish, material composition, flatness, and thermal properties affect the measured length. The surface finish and material composition affect the phase shift and the flatness affects the wringing layer between the block and platen. The mechanical comparisons are not affected by any of these factors. The major remaining factor is the thermal expansion. We therefore pool the control data for similar size blocks. Each group has about 20 sizes, until the block lengths become greater than 25 mm. For these blocks the thermal differences are very small. For longer blocks, the temperature effects become dominant and each size represents a slightly different process; therefore the data are not combined.

For this analysis we break down the reproducibility into three regimes: thin blocks (less than 1 mm), long blocks (>100 mm), and the intermediate range that contains most of the blocks we measure. This is a natural breakdown because blocks ≤100 mm are measured with a different type of comparator and a different comparison scheme than are used for blocks >100 mm. A fit to the historical data produces an uncertainty component (standard deviation) for each group as shown in Table 5.

### 5.3 Thermal Expansion

#### 5.3.1 Thermometer Calibration

For comparison measurements of similar materials, the thermometer calibration is not very important since the temperature error is the same for both blocks.

#### 5.3.2 Coefficient of Thermal Expansion

The variation in the CTE for similar gage block materials is generally smaller than the ±1×10^−6^/°C allowed by the ISO and ANSI gage block standards. From the variation of our own steel master blocks, we estimate the standard uncertainty of the CTE to be 0.4×10^−6^/°C. Since we do not measure gage blocks if the temperature is more than 0.2 °C from 20 °C, the length-standard uncertainty is 0.08×10^−6^ *L*. For long blocks (*L*>100 mm) we do not perform measurements if the temperature is more than 0.1 °C from 20 °C, reducing the standard uncertainty to 0.04×10^−6^ *L*.

#### 5.3.3 Thermal Gradients

The uncertainty due to thermal gradients is important. For the short block comparator temperature differences up to ±0.030 °C have been measured between blocks positioned on the comparator platen. Assuming a rectangular distribution we get a standard temperature uncertainty of 0.017 °C. The temperature difference affects the entire length of the block, and the length standard uncertainty is the temperature difference times the CTE times the length of the block. Thus for steel it would be 0.20×10^−6^ *L* and for chrome carbide 0.14×10^−6^ *L*. For our simplified discussion here we use the average value of 0.17×10^−6^ *L*.

The precautions used for long block comparisons result in much smaller temperature differences between blocks, 0.010 °C and less. Using this number as the half-width of a rectangular distribution we get a standard temperature uncertainty of 0.006 °C. Since nearly all blocks over 100 mm are steel we find the standard uncertainty component to be 0.07×10^−6^ *L*.

### 5.4 Elastic Deformation

Since most of our calibrations compare blocks of the same material, the elastic deformation corrections are not needed. There is, in theory, a small variability in the elastic modulus of blocks of the same material. We have not made systematic measurements of this factor. Our current comparators have nearly flat contacts, from wear, and we calculate that the total deformations are less than 0.05 μm. If we assume that the elastic properties of gage blocks of the same material vary by less than 5 % we get a standard uncertainty of 0.002 μm. We have tested ceramic blocks and found that the deformation is the same as steel for our conditions.

For materials other than steel, chrome carbide, and ceramic (zirconia), we must make penetration corrections. Unfortunately, we have discovered that the diamond styli wear very quickly and the number of measurements which can be made without measurable changes in the contact geometry is unknown. From our historical data, we know that after 5000 blocks, both of our comparators had flat contacts. We currently add an extra component of uncertainty for measurements of blocks for which we do not have master blocks of matching materials.

### 5.5 Scale Calibration

The gage block comparators are two point-contact devices, the block being held up by an anvil. The length scale is provided by a calibrated linear variable differential transformer (LVDT). The LVDT is calibrated *in situ* using a set of gage blocks. The blocks have nominal lengths from 0.1 in to 0.100100 in with 0.000010 in steps. The blocks are placed between the contacts of the gage block comparator in a drift eliminating sequence; a total of 44 measurements, four for each block, are made. The known differences in the lengths of the blocks are compared with the measured voltages and a least-squares fit is made to determine the slope (length/voltage) of the sensor. This calibration is done weekly and the slope is recorded. The standard deviation of this slope history is taken as the standard uncertainty of the sensor calibration, i.e., the variability of the scale magnification. Over the last few years the relative standard uncertainty has been approximately 0.6 %. Since the largest difference between the customer and master block is 0.4 μm (from customer histories), the standard uncertainty due to the scale magnification is 0.006×0.4 μm = 0.0024 μm.

The long block comparator has older electronics and has larger variability in its scale calibration. This variability is estimated to be 1 %. The long blocks also have a much greater range of values, particularly blocks manufactured before the redefinition of the in in 1959. When the in was redefined its value changed relative to the old in by 2×10^−6^, making the length value of all existing blocks larger. The difference between our master blocks and customer blocks can be as large as 2 μm, and the relative standard uncertainty of 1 % in the scale linearity yields a standard uncertainty of 0.020 μm.

### 5.6 Instrument Geometry

If the measurements are comparisons between blocks with perfectly flat and parallel gaging surfaces, the uncertainties resulting from misalignment of the contacts and anvil are negligible. Unfortunately, the artifacts are not perfect. The interaction of the surface flatness and the contact alignment is a small source of variability in the measurements, particularly for thin blocks. Thin blocks are often warped, and can be out of flat by 10 μm, or more. If the contacts are not aligned exactly or the contacts are not spherical, the contact points with the block will not be perpendicular to the block. Thus the measurement will be slightly larger than the true thickness of the block. We have made multiple measurements on such blocks, rotating the block so that the angle between the block surface and the contact line varies as much as possible. From these variations we find that for thin blocks (<1 mm), the standard uncertainty is 0.010 μm.

### 5.7 Artifact Geometry

The definition of length for a gage block is the perpendicular distance from the gage point on top to the corresponding point on the flat surface (platen) to which it is wrung. If the platen and gage block are perfectly flat this distance would be the mechanical distance from the gage points on the top and bottom of the block plus the thickness of the wringing layer. If the customer block also was perfectly flat, the difference in the defined length (from interferometry) and the mechanical length (from the two-point comparison) would be the same.

The customer block and the NIST master are not, of course, perfectly flat. This leaves the possibility that the calibration will be in error because the comparison process, in effect, assigns the bottom geometry and wringing film of the NIST master to the customer block.

We have attempted to estimate this error from our history of the measurements of the 2 mm series of metric blocks. All of these blocks are steel and from the same manufacturer, eliminating the complications of the interferometric phase correction. If there is no error due to surface flatness, the length difference found by interferometry and by mechanical comparisons should be equal.

Analyzing this data is difficult. Since either or both of the blocks could be the cause of an offset, the average offset seen in the data is expected to be zero. The signature of the effect is a wider distribution of the data than expected from the individual uncertainties in the interferometry and comparison process.

For each size the difference between interferometric and mechanical length is a measure of the bias caused by the geometry of the gaging surfaces of the blocks. This bias is calculated from the formula

$$B=\left(L1_{\mathrm{int}}-L2_{\mathrm{int}}\right)-\left(L1_{\text{mech}}-L2_{\text{mech}}\right)$$ (11)

where *B* is the bias, *L*1_int_ and *L*2_int_ are the lengths of blocks 1 and 2 measured by interferometry, and *L*1_mech_ and *L*2_mech_ the lengths of blocks 1 and 2 measured by mechanical comparison. Because the geometry effects can be of either sign, the average bias over a number of blocks is zero. There is, fortunately, more useful information in the variation of the bias because it is made up of three components: the variations in the interferometric length, the mechanical length, and the geometry effects. The variation in the interferometric and mechanical length differences can be obtained from the interferometric history and the check standard data, respectively. Assuming that all of the distributions are normal, the measured standard deviations are related by:

$$S_{\text{bias}}^{2}=S_{\mathrm{int}}^{2}=S_{\text{mech}}^{2}=S_{\text{geom}}^{2}$$ (12)

Our data for the 2 mm series is shown below. The numbers given are somewhat different than the tables show for typical calibrations for these sizes. The 2 mm series is not very popular with our customers, and since we do few calibrations in these sizes there are fewer interferometric measurements of the masters and fewer check standard data. We analyzed 58 pairs of blocks from the 2 mm series blocks and obtained estimated standard deviations of 0.017 μm for the bias, 0.014 μm for the interferometric differences and 0.005 μm for the mechanical differences. This gives 0.008 μm as the standard uncertainty in gage length due to the block surface geometry.

Another way to estimate this effect is to measure the blocks in two orientations, with each end wrung to the platen in turn. We have not made a systematic study with this method but we do have some data gathered in conjunction with international interlaboratory tests. This data suggest that the effect is small for blocks under a few millimeters, but becomes larger for longer blocks. This suggests that the thin blocks deform to the shape of the plated when wrung, but longer blocks are stiff enough to resist the deformation. Since both of the surfaces are made with the same lapping process, this estimate may be somewhat smaller than the general case. This effect is potentially a major source of uncertainty and we plan further tests in the future.

### 5.8 Summary

The uncertainty budget for gage block calibration by mechanical comparison is shown in Table 6. The expanded uncertainty (coverage factor *k* = 2) for each type of calibration is

**Table:** 

Thin Blocks (*L*<1 mm)	*U*=0.040 μm
Gage Blocks (1 mm to 100 mm)	*U*=0.030 μm+0.35×10^−6^ *L*
Long Blocks (100mm<*L*≤500 mm)	*U*=0.055 μm+0.20×10^−6^ *L*.

For long blocks with known thermal expansion coefficients, the uncertainty is smaller than stated above.

### 6. Gage Wires (Thread and Gear Wires) and Cylinders (Plug Gages)

Customer wires are calibrated by comparison to master wires using several different micrometers. Most of the micrometers have flat parallel contacts, but cylinder contacts are occasionally used. The sensors are mechanical twisted thread comparators, electronic LVDTs, and interferometers.

For gage wires, gear wires, and cylinders we report the undeformed diameter. If the wire is a thread wire the proper deformation is calculated and the corrected (deformed) diameter is reported.

The master wires and cylinders have been calibrated by a variety of methods over the last 20 years:

1. Large cylinders are usually calibrated by comparison to gage blocks using a micrometer with flat contacts.
2. A second device uses two optical flats with gage blocks wrung in the center as anvils. The upper flat has a fixture that allows it to be set at any height and adjusted nearly parallel to the bottom flat. The wire or cylinder is placed between the two gage blocks (wrung to the flats) and the top flat is adjusted to form a slight wedge. This wedge forms a Fizeau interferometer and the distance between the two flats is determined by multicolor interferometry. The cylinder or wire diameter is the distance between the flats minus the lengths of the gage blocks.
3. A third device consists of a moving flat anvil and a fixed cylindrical anvil. A displacement interferometer measures the motion of the moving anvil.
4. Large diameter cylinders can be compared to gage blocks using a gage block comparator.

### 6.1 Master Artifact Calibration

The master wires are measured by a number of methods including interferometry and comparison to gage blocks. We will take the uncertainty in the wires and cylinders as the standard deviation of the master calibrations over the last 20 years. Because of the number of different measurement methods, each with its own characteristic systematic errors, and the long period of time involved, we assume that all of the pertinent uncertainty sources have been sampled. The standard deviation derived from 168 degrees of freedom is 0.065 μm.

### 6.2 Long Term Reproducibility

**Fig. 6:**
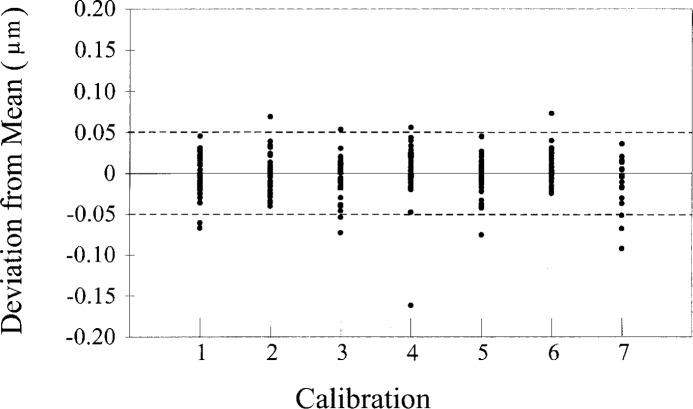
Check standard data for seven calibrations of a set of thread wires.

We use check standards extensively in our wire calibrations, which produces a record of the long term reproducibility of the calibration. A typical data set is shown in Fig. 6.

While we do not use check standards for every size and type of wire, the difference in the measurement process for similar sizes is negligible. From our long-term measurement data we find the standard uncertainty for reproducibility (one standard deviation, 300 degrees of freedom) is *u* = 0.025 μm.

### 6.3 Thermal Expansion

#### 6.3.1 Thermometer Calibration

Since all customer calibrations are done by mechanical comparison the uncertainty due to the thermometer calibration is negligible.

#### 6.3.2 Coefficient of Thermal Expansion

Nearly all wires are steel, although some cylinders are made of other materials. Since we measure within 0.2 °C of 20 °C, if we assume a rectangular distribution and we we know the CTE to about 10 %, we get a differential expansion uncertainty of 0.01×10^−6^ *L*.

#### 6.3.3 Thermal Gradients

We have found temperature differences up to ±0.030 °C in the calibration area of our comparators, and using 0.030 °C as the half-width of a rectangular distribution we get a standard temperature uncertainty of 0.017 °C, which leads to a length standard uncertainty of 0.017×10^−6^ *L*.

### 6.4 Elastic Deformation

The elastic deformations under the measurement conditions called out in the screw thread standard [10] are very large, 0.5 μm to 1 μm. Because we do not perform master wire calibrations at standard conditions we must make corrections for the actual deformation during the measurement to get the undeformed diameter, and then apply a further correction to obtain the diameter at the standard conditions. When both deformation corrections are applied to the master wire diameter, the comparator process automatically yields the correct standard diameter of the customer wires.

Our corrections are calculated according to formulas derived at Commonwealth Scientific and Industrial Research Organization (CSIRO), and have been checked experimentally. There is no measurable bias between the calculated and measured deformations when the elastic modulus of the material is well known. Unfortunately, there is a significant variation in the reported elastic modulus for most common gage materials. An examination of a number of handbooks for the elastic moduli gives a relative standard deviation of 3 % for hardened steel, and 5 % for tungsten carbide.

**Fig. 7:**
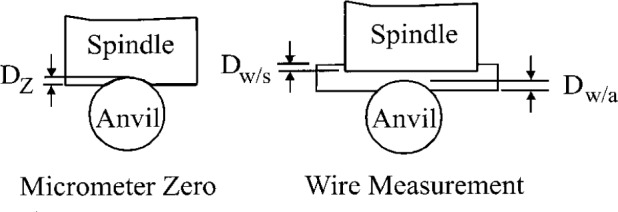
Schematic depiction of the measurement of the wire/spindle and spindle/anvil contacts are line contacts and involve small deformations. The wire/anvil contact is a point contact and the deformation is large.

If we examine a typical case for thread wires (40 pitch) we have the corrections shown in Fig. 7. Line contacts have small deformations and point contacts have large deformations. For a typical wire calibration the deformation at the micrometer zero, *Dz*, is a line contact with a deformation of 0.003 μm. The deformation of the wire at the micrometer flat contact, *Dw*/*s*, is also a line contact with a value of 0.003 μm. The contact between the micrometer cylinder anvil and wire is a point contact, *Dw*/*a*, which has the much larger deformation of 0.800 μm.

Once these corrections are made the wire measurement is the undeformed diameter. To bring the reported diameter to the defined diameter (deformed at ASME B1 conditions) a further correction of 1.6 μm must be made. The corrections are thus from a slightly deformed diameter, as measured, to the undeformed diameter, and from the undeformed diameter to the standard (B1) deformed diameter. Since all of the corrections use the same formula, which we have assumed is correct, the only uncertainty is in the difference between the two corrections. In our example this is 0.8 μm.

Nearly all gage wires are made of steel. If we take the elastic modulus distribution of steel to be rectangular with a half-width of 3 % and apply it to this differential correction, we get a standard uncertainty of 0.013 μm.

### 6.5 Scale Calibration

The comparator scales are calibrated with gage blocks. Since several different comparators are used, each calibrated independently with different gage blocks, the check standard data adequately samples the variability in sensor calibration.

### 6.6 Instrument Geometry

When wires and cylinders are calibrated by comparison, the instrument geometry is the same for both measurements and thus any systematic effects are the same. Since the difference between the measurements is used for the calibration, the effects cancel.

### 6.7 Artifact Geometry

If the cylinder is not perfectly round, each time a different diameter is measured the answer will be different. We measure each wire or cylinder multiple times, changing the orientation each time. If the geometry of the wire is very bad this variation in the readings will cause the calibration to fail the control test for repeatability. If not, the wire will pass and the uncertainty assigned will be from the check standard data, i.e., the check standard wires. Since we pool check standard data from a number of similar size wires, the check standard data includes the effects of “average” roundness.

If the customer wire is significantly more out of round than our check standard the calibration will fail the repeatability test. In these cases we increase the reported uncertainty. For customers who need the highest accuracy, we measure the roundness as part of the calibration or make measurements only along one marked diameter. The customer then makes measurements using the same diameter.

Wires and cylinders can also be tapered. Since we measure the wires in the middle, but fixture the wires by hand, there is some uncertainty in the position of the measurement. According to the thread wire standard, wires must be tapered less than 0.254 μm (10 μin) over the central 25 mm (1 in) of their length. Most of the wires we calibrate are master wires and are much better than this limit. Since the fixturing error is less than a few millimeters, the resultant uncertainty in diameter is small. As an estimate we assume the central 25 mm of the wire has a possible diameter change of 0.1 μm, giving a possible diameter change of 0.008 μm for an assumed 2 mm fixturing shift.

### 6.8 Summary

Table 7 shows the uncertainty budget for wire and cylinder calibrations. We combine the standard uncertainties and use a coverage factor of *k* = 2 to obtain the expanded uncertainty *U* for wires up to 25 mm in diameter of *U* = 0.14 μm+0.03×10^−6^ *L*.

The cylinder calibrations are done with little or no deformation and therefore the last uncertainty source, elastic deformation, is negligible. The change is not, however, large enough to change the uncertainty in the second decimal digit.

## 7. Ring Gages (Diameter <100 mm)

Ring gages are measured by comparison to one or more gage block stacks. Most ring gages have a marked diameter and this particular diameter is the only reported value.

### 7.1 Master Artifact Calibration

**Fig. 8:**
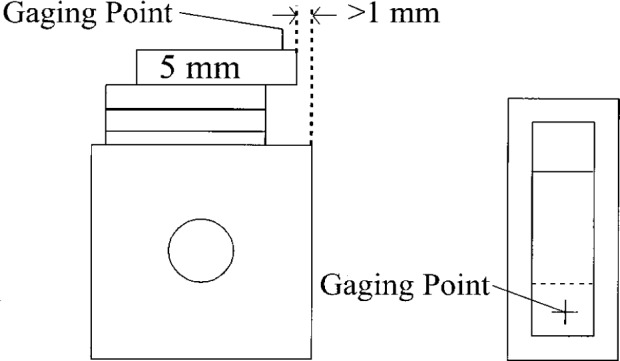
Schematic depiction of the use of a gage block stack for use as a master gage for ring gage calibration.

Ring gages, in general, do not come in sets of standardized sizes. Because of this we cannot have master ring gages. Instead we calibrate ring gages by comparison to a stack of gage blocks wrung to a precision square. A gage block stack is prepared the same length as the nominal ring diameter. This stack is wrung to a precision square and a 5 mm block is wrung on top so that about 10 mm of the block extend beyond the end of the stack, as shown in Fig. 8. This extended surface of the top block and the surface of the square forms an internal length to compare with the ring.

The square is longer and wider than the gage block stack so that the fringe fractions between the surface of the square and the top of the gage block stack are clearly visible. The quality of the wring can be seen by examining the fringes. If the fringes on the block stack and square are parallel and straight the wring is good.

Each stack is measured by multicolor interferometry to give the highest possible accuracy. The gap is the difference between the measured length of the top block and the length of the entire stack. Since the length of the top block is measured on the square, there is no phase correction needed, reducing the uncertainty. As an estimate we use the uncertainty of customer block calibrations without the phase correction uncertainty. Since the gap is the difference of two measurements the total uncertainty is the root-sum-square of two measurements. The standard uncertainty is 0.038 μm+0.2×10^−6^ *L*.

### 7.2 Long Term Reproducibility

The ring gages we calibrate come in a large variety of sizes and it is impractical to have master ring gages and check standards. We do not calibrate enough rings of any one size to generate statistically significant data. As an alternative, we use data from our repeat customers with enough independent measurements on the same gages to estimate our long term reproducibility. Measurements on four gages from one customer over the last 20 years show a standard deviation of 0.025 μm.

### 7.3 Thermal Expansion

#### 7.3.1 Thermometer Calibration

The thermometer calibration affects the length of the master stack, but this effect has been included in the uncertainty of the stack (master).

#### 7.3.2 Coefficient of Thermal Expansion

We choose gage blocks of the same material as the customer gage to make the master gage block stack. This reduces the differential expansion coefficient. By using similar materials as test and master gage the standard uncertainty of the differential thermal expansion coefficient is 0.6×10^−6^/°C and all of the measurements are made within 0.2 °C of 20 °C. This uncertainty in thermal expansion coefficient gives a length standard uncertainty of 0.2×0.6×10^−6^ *L*, or 0.12×10^−6^ *L*.

#### 7.3.3 Thermal Gradients

We have measured the temperature variation of the ring gage comparator and found it is generally less than 0.020 °C. Using steel as our example, the possible temperature difference between gages produces a proportional change in the ring diameter Δ*L*/*L* of (11.5×10^−6^)×(0.020 °C) = 0.23×10^−6^. Since our reproducibility includes a number of measurements in different years, and thus different conditions, this component of uncertainty is sampled in the reproducibility data and is not considered as a separate component of uncertainty.

### 7.4 Elastic Deformation

Since the master gage and ring are of the same material the elastic deformation corrections are nearly the same. There is a small correction because in one case the contact is a probe ball against a plane and in the ring case the ball probe is against a cylinder. These corrections, however, are less than 0.050 μm. Since we make the corrections, the only uncertainty is associated with our knowledge of the elastic modulus and Poisson’s Ratio of the materials. Using 5 % as the relative standard uncertainty of the elastic properties, we get a standard uncertainty in the elastic deformation correction of 0.005 μm.

### 7.5 Scale Calibration

The ring comparator is calibrated using two or more calibrated gage blocks. Since the uncertainty of these blocks is less than 0.030 μm, and the comparator scale is 2.5 μm, the uncertainty in the slope is about 1 % (95 % confidence level). The difference between the ring and gage block stack wrung as the master is less than 0.5 μm, leading to a standard uncertainty of 0.5 % of 0.5 μm, or 0.0025 μm.

### 7.6 Instrument Geometry

The master and gage are manipulated to assure that alignment errors are not significant. The ring is moved small amounts until the readings are maximized, and the maximum diameter is recorded. The gage block stack is rotated slowly to minimize its reading. Since both errors are cosine errors this procedure is fairly simple. Another error is the squareness of the flat reference surface of the ring to its cylinder axis. This alignment is tested separately and corrections are applied as needed.

The remaining source of error is the alignment of the contacts. If the relative motion of the two contacts is parallel but not coincident, the transfer of length from the gage block stack (with flat parallel surfaces) to the ring gage (cylindrical surface) will have an error which is proportional to the square of the distance the two sensor axes are displaced. We have tested for this error using very small diameter cylinders and have found no effect at the 0.025 μm level. This provides a bound on the axis displacement of 5 μm. This level displacement would produce possible errors in wring calibrations of up to 0.020 μm on 3 mm rings and proportionately smaller errors on larger diameter rings. If we assume the 0.020 μm represents the half-width of a rectangular distribution, we get a standard uncertainty of 0.012 μm for 3 mm rings. Since we rarely calibrate a ring with a diameter under 5 mm, we take 0.010 μm as our standard uncertainty.

### 7.7 Customer Artifact Geometry

Ring gages have a marked diameter and we measure only this diameter. The roundness of the ring does not affect the measurement. We do provide roundness traces of the ring on customer request.

### 7.8 Summary

The uncertainty budget for ring gage calibration is shown in Table 8. The expanded uncertainty *U* for ring gages up to 100 mm diameter (*k* = 2) is *U* = 0.094 μm+0.36×10^−6^ *L*.

## 8. Gage Balls (Diameter)

Gage balls are measured directly by interferometry or by comparison to master balls using a precision micrometer. The interferometric measurement is made by having the ball act as the spacer between two coated optical flats or an optical flat and a steel platen. The flats are fixtured so that they can be adjusted nearly parallel, forming a wedge. The fringe fraction is read at the center of the ball for each of four colors and analyzed in the same manner as multi-color interferometry of gage blocks. A correction is applied for the deformation of the flats in contact with the balls and when the steel platen is used, and for the phase change of light on reflection from the platen.

### 8.1 Master Artifact Calibration

Master balls are calibrated by interferometry, using the ball as a spacer in a Fizeau interferometer or by comparison to gage blocks. The master ball historical data covers a number of calibration methods over the last 30 years. An analysis of this data gives a standard deviation of 0.040 μm with 240 degrees of freedom. Since these measurements span a number of different types of sensors, multiple sensor calibrations, systematic corrections, and environmental corrections, there are very few sources of variation to list separately. The only significant remaining sources are the uncertainties of the frequencies of the cadmium spectra, which are negligible for the typical balls (<30 mm) calibrated by interferometry. We take the standard deviation of the measurement history as the standard uncertainty of the master balls.

### 8.2 Long Term Reproducibility

The long term reproducibility of gage ball calibration was assessed by collecting customer data over the last 10 years. The standard deviation, with 128 degrees of freedom is found to be 0.035 μm. There is no evident length dependence because there are very few gage balls over 30 mm in diameter. For large balls the uncertainty is derived from repeated measurements on the gage in question.

### 8.3 Thermal Expansion

#### 8.3.1 Thermometer Calibration

Gage balls are measured by comparison to the master balls. Since our master balls are steel, there is little uncertainty due to the thermometer calibration for the calibration of steel balls. This is not true for other materials. Tungsten carbide is the worst case. For a thermometer calibration standard uncertainty of 0.01 °C, we get a standard uncertainty from the differential expansion of steel and tungsten carbide of 0.08×10^−6^ *L*.

#### 8.3.2 Coefficient of Thermal Expansion

We take the relative standard uncertainty in the thermal expansion coefficients of balls to be the same as for gage blocks, 10 %. Since our comparison measurements are always within 0.2 °C of 20 °C the standard uncertainty in length is 1×10^−6^/°C×0.2 °C×*L* = 0.2×10^−6^ *L*.

#### 8.3.3 Thermal Gradients

We have found temperature differences up to 0.030 °C between balls, which would lead to a standard uncertainty of 0.3×10^−6^ *L*. Using ±0.030×10^−6^ *L* as the span of a rectangular distribution we get a standard uncertainty of 0.17×10^−6^ *L*.

### 8.4 Elastic Deformation

There are two sources of uncertainty due to elastic deformation. The first is the correction applied when calibrating the master ball. For balls up to 25 mm in diameter the corrections are small and the major source of uncertainty is from the uncertainty in the elastic modulus. If we assume 5 % relative standard uncertainty in the elastic modulus, the standard uncertainty in the deformation correction is 0.010 μm.

The second source is from the comparison process. If both the master and customer balls are of the same material, then no correction is needed and the uncertainty is negligible. If the master and customer balls are of different materials, we must calculate the differential deformation. The uncertainty of this correction is also due to uncertainty of the elastic modulus. While the uncertainty of the difference between the elastic properties of the two balls is greater than for one ball, the differential correction is smaller than for the absolute calibration of one ball, and the standard uncertainty remains nearly the same, 0.010 μm.

### 8.5 Scale Calibration

The comparator scale is calibrated with a set of gage blocks of known length difference. Since the range of the comparator is 2 μm and the block lengths are known to 0.030 μm, the slope is known to approximately 1 %. Customer blocks are seldom more than 0.3 μm from the master ball diameter, so the uncertainty is less than 0.003 μm.

### 8.6 Instrument Geometry

The flat surfaces of the comparator are parallel to better than 0.030 μm. Since the balls are identically fixtured during the measurements, there is negligible error due to surface flatness. The alignment of the scale with the micrometer motion produces a cosine error, which, given the very small motion, is negligible.

### 8.7 Artifact Geometry

The reported diameter of a gage ball is the average of several measurements of the ball in random orientations. This means that if the customer ball is not very round, the reproducibility of the measurement is degraded. For customer gages suspected of large geometry errors we will generally rotate the ball in the micrometer to find the range of diameters found. In some cases roundness traces are performed. We adjust the assigned uncertainty for balls that are significantly out of round.

### 8.8 Summary

From Table 9 it is obvious that the length-dependent terms are too small to have a noticeable affect on the total uncertainty. For customer artifacts that are significantly out-of-round, the uncertainty will be larger because the reproducibility of the comparison is affected. For these and other unusual calibrations, the standard uncertainty is increased. The expanded uncertainty *U* (*k* = 2) for balls up to 30 mm in diameter is *U* = 0.11 μm.

## 9. Roundness Standards (Balls, Rings, etc.)

Roundness standards are calibrated on an instrument based on a very high accuracy spindle. A linear variable differential transformer (LVDT) is mounted on the spindle, and is rotated with the spindle while in contact with the standard. The LVDT output is monitored by a computer and the data is recorded. The part is rotated 30° 11 times and measured in each of the orientations. The data is then analyzed to yield the roundness of the standard as well as the spindle. The spindle roundness is recorded and used as a check standard for the calibration.

### 9.1 Master Artifact Calibration

The roundness calibration is made using a multiple-redundant closure method [21] and does not require a master artifact.

### 9.2 Long Term Reproducibility

Data from multiple calibrations of the same roundness standards for customers were collected and analyzed. The data included measurements of six different roundness standards made over periods as long as 15 years. The standard deviation of a radial measurement, derived from this historical data (60 degrees of freedom), is 0.008 μm.

### 9.3 Thermal Expansion

Measurements are made in a temperature controlled environment (±0.1 °C) and care is taken to allow gradients in the artifact caused by handling to equilibrate. The roundness of an artifacts is not affected by homogeneous temperature changes of the magnitude allowed by our environmental control.

### 9.4 Elastic Deformation

Since the elastic properties of the artifacts are homogeneous the probe deformations are also homogeneous and thus irrelevant.

### 9.5 Sensor Calibration

The LVDT is calibrated with a magnification standard. At our normal magnification for roundness calibrations the magnification standard uncertainty is approximately 0.10 μm over a 2 μm range. Since most roundness masters calibrated in our laboratory have deviations of less than 0.03 μm, the standard uncertainty due to the probe calibration is less than 0.002 μm.

### 9.6 Instrument Geometry

The closure method employed measures the geometrical errors of the instrument as well as the artifact and makes corrections. Thus only the non-reproducible geometry errors of the instrument are relevant, and these are sampled in the multiple measurements and included in the reproducibility standard deviation.

### 9.7 Customer Artifact Geometry

For roundness standards with a base, the squareness of the base to the cylinder axis is important. If this deviates from 90° the cylinder trace will be an ellipse. Since the eccentricity of the trace is related to the cosine of the angular error, there is generally no problem. Our roundness instrument has a *Z* motion (direction of the cylinder axis) of 100 mm and is straight to better than 0.1 μm. It is used to check the orientation of the standard in cases where we suspect a problem.

For sphere standards a marked diameter is usually measured, or three separate diameters are measured and the data reported. Thus there are no specific geometry-based uncertainties.

### 9.8 Summary

Table 10 gives the uncertainty budget for calibrating roundness standards. Since the thermal and scale uncertainties are negligible, the only major source of uncertainty is the long term reproducibility of the calibration. Using a coverage factor *k* = 1 the expanded uncertainty *U* of roundness calibrations is *U* = 0.016 μm.

## 10. Optical Flats

Optical flats are calibrated by comparison to calibrated master flats. The master flats are calibrated using the three-flat method, which is a self-calibrating method [22]. In the three flat method only one diameter is calibrated. For our customer calibrations the test flat is measured and then rotated 90° so that a second diameter can be measured.

The test flat is placed on top of the master flat, supported by three thin spacers placed 0.7 times the radius from the center at 120° angles from each other. The master flat is supported on a movable carriage in a similar (three point) manner. These supports assure that the measured diameter of both flats are undeformed from their free state. For metal or partially coated reference flats the test flat is place on the bottom and the master flat placed on top.

One of the three spacers between the flats is slightly thicker than the other two, making the space between the flats a wedge. When this wedge is illuminated by monochromatic light, distinct fringes are seen. The straightness of these fringes corresponds to the distance between the flats, and is measured using a Pulfrich viewer [23].

### 10.1 Master Artifact Calibration

The master flat is calibrated with the same apparatus used for customer calibrations, the only difference being that for a customer calibration the customer flat is compared to a master flat, and for master flat calibrations, the master flat is compared with two other master flats of similar size. Sources of uncertainty other than the long term reproducibility of the comparison measurement are negligible (see Secs. 11.3 to 11.7).

The actual three flat calibration of the master flat uses comparisons of all three flats against each other in pairs. The contour is measured on the same diameter on each flat for all of the combinations. The first measurement using flats A and B is

$$m_{\text{AB}}\left(\chi \right)=F_{A}\left(\chi \right)+F_{B}\left(\chi \right),$$ (13)

where *F* (*χ*) is the variation in the height of the air layer between the two flats. The value is positive when the surface is outside of the line connecting the endpoints (i.e., a convex flat has *F* (*χ*) positive everywhere). Flat C replaces flat B and the contour along the same diameter is remeasured:

$$m_{\text{AC}}\left(\chi \right)=F_{A}\left(\chi \right)+F_{C}\left(\chi \right)$$ (14)

Flat B is placed on the bottom and C on top and the contour is measured.

$$m_{\text{BC}}\left(\chi \right)=F_{B}\left(\chi \right)+F_{C}\left(\chi \right).$$ (15)

The shape of flat A is then

$$F_{A}\left(\chi \right)=\frac{1}{2}\left[m_{\text{AB}}\left(\chi \right)+m_{\text{AC}}\left(\chi \right)-m_{\text{BC}}\left(\chi \right)\right]$$ (16)

Since all three measurements use the same procedure the uncertainties are the same. If we denote the standard uncertainty of one flat comparison as *u*, the standard uncertainty *u*_A_ in *F*_A_(*χ*) is related to *u* by

$$u_{A}=\sqrt{\frac{3u^{2}}{4}.}$$ (17)

Thus the standard uncertainty of the master flat is the square root of 3/4 or about 0.9 times the standard uncertainty of one comparison.

To estimate the long term reproducibility, we have compared calibrations of the same flat using two different master flats over an eight year period. This comparison shows a standard deviation (60 degrees of freedom) of 3.0 nm. Using this value in Eq. (16) we find the standard uncertainty of the master flat to be 0.0026 μm.

### 10.2 Long Term Reproducibility

As noted above, for a customer flat the standard uncertainty of the comparison to the master flat is 0.003 μm.

### 10.3 Thermal Expansion

The geometry of optical flats is relatively unaffected by small homogeneous temperature changes. Since the calibrations are done in a temperature controlled environment (±0.1 °C), there is no correction or uncertainty related to temperature effects.

### 10.4 Elastic Deformation

The flatness of the surface of an optical flat depends strongly on the way in which it is supported. Our calibration report includes a description of the support points and the uncertainty quoted applies only when the flat is supported in this manner. Changing the support points by small amounts (1 mm or less, characteristic of hand placement of the spacers) produces negligible changes in surface flatness.

### 10.5 Sensor Calibration

The basic scale of the measurement is the wavelength of light. For optical flats the fringe straightness is smaller than the fringe spacing, and is measured to about 1 % of the fringe spacing. Thus the wavelength of the light need only be known to better than 1 %. Since a helium lamp is used for illumination, even if the index of refraction corrections are ignored the wavelength is known with an uncertainty that is a few orders of magnitude smaller than needed.

### 10.6 Instrument Geometry

The flats are transported under the viewer on a one dimensional translation stage. Since the fringes are less than 5 mm apart and are measured to about 1 % of a fringe spacing, as long as the straightness of the waybed motion is less than 5 μm the geometry correction is negligible. In fact, the waybed is considerably better than needed.

### 10.7 Customer Artifact Geometry

There are no test artifact-related uncertainty sources.

### 10.8 Summary

Table 11 shows the uncertainty budget for optical flat calibration. The only non-negligible uncertainty source is the master flat and the comparison reproducibility. The expanded uncertainty *U* (*k* = 2) of the calibration is therefore *U* = 0.008 μm.

## 11. Indexing Tables

Indexing tables are calibrated by closure methods using a NIST indexing table as the second element and a calibrated autocollimator as the reference [24]. The customer’s indexing table is mounted on a stack of two NIST tables. A plane mirror is then mounted on top of the customer table. The second NIST table is not part of the calibration but is only used to conveniently rotate the entire stack.

Generally tables are calibrated at 30° intervals. Both indexing tables are set at zero and the autocollimator zeroed on the mirror. The customer’s table is rotated clockwise 30° and our table counter-clockwise 30°. The new autocollimator reading is recorded. This procedure is repeated until both tables are again at zero.

The stack of two tables is rotated 30°, the mirror repositioned, and the procedure repeated. The stack is rotated until it returns to its original position. From the readings of the autocollimator the calibration of both the customer’s table and our table is obtained. The calibration of our table is a check standard for the calibration.

### 11.1 Master Artifact Calibration

As discussed above there is no master needed in a closure calibration.

### 11.2 Long Term Reproducibility

Each indexing calibration produces a measurement repeatability for the procedure. Our normal calibration uses the closure method, comparing the 30° intervals of the customer’s table with one of our tables. One of the 30° intervals may be subdivided into six 5° subintervals, and one of the 5° subintervals may be subdivided into 1° subintervals. The method of obtaining the standard deviation of the intervals is documented in NBSIR 75-750, “The Calibration of Indexing Tables by Subdivision,” by Charles Reeve [24]. Since each indexing table is different and may have different reproducibilities we use the data from each calibration for the uncertainty evaluation.

As an example and a check on the process, we have examined the data from the repeated calibration of the NIST indexing table used in the calibration. Six calibrations over a 10 year span show a pooled standard deviation of 0.07*″* for 30° intervals. The average uncertainty (based on short term repeatability of the closure procedure) for each of the calibrations is within round-off of this value, showing that the short and long term reproducibility of the calibration is the same.

### 11.3 Thermal Expansion

The calibrations are performed in a controlled thermal environment, within 0.1 °C of 20 °C. Temperature effects on indexing tables in this environment are negligible.

### 11.4 Elastic Deformation

There is no contact with the sensors so there is no deformation caused by the sensor. There is deformation of the indexing table teeth each time the table is repositioned. This effect is a major source of variability in the measurement, and is adequately sampled in the procedure.

### 11.5 Sensor Calibration

The autocollimators are calibrated in a variety of ways, including differential motions of stacked indexing tables, reversal of angle blocks (typically 1*″* and 5*″*), precision angle generators, sine plates and comparison with commercial laser interferometer based angle measurement systems. The uncertainty in generating a 10*″* angle change by any of these methods is small.

Since the high quality indexing tables calibrated at NIST typically have deviations from nominal of less than 2*″*, the uncertainty component related the autocollimator calibration is negligible on the order of 0.01*″*, which is negligible.

### 11.6 Instrument Geometry

There are several subtle problems due to the flatness of the reference mirror and alignment of the two indexing tables that affect the calibration. However, with proper alignment of the table and mirror, the autocollimator will illuminate the same area of the mirror for each measurement. This eliminates the effects of the mirror flatness on the measurement.

### 11.7 Customer Artifact Geometry

The rotational errors (runout, tilt) of the typical indexing table are too small to have a measurable effect on the measurement.

### 11.8 Summary

Table 12 shows the uncertainty budget for indexing table calibrations. The expanded uncertainty *U* (*k* = 2) of indexing table calibrations is estimated to be *U* = 0.14*″*.

## 12. Angle Blocks

Angle blocks are calibrated by comparison to master angle blocks using an angle block comparator. The angle block comparator consists of two high accuracy autocollimators and a fixture which allows angle blocks of the same size to be positioned repeatably in the measurement paths of the autocollimators. The autocollimators are adjusted to zero on the surfaces of the master angle block, and then the customer angle block is substituted for the master. Customer angle blocks, the master angle block, and a check standard are each measured multiple times. The changes in the autocollimator readings are recorded and analyzed to yield the angles of the customer blocks, the angle of the check standard, and the standard deviation of the comparison scheme. The latter two items of data are used as statistical process control parameters.

### 12.1 Master Artifact Calibration

The master angle blocks are measured by a number of methods depending on their angle. Angle blocks of nominal angle 1*′* or less can be calibrated using an indexing table and autocollimator by simple reversal. The 15° and larger blocks are calibrated by closure methods related to the indexing table calibration. In these methods the angle of the angle block is compared with similar angles of the indexing table. For example, a 90° angle block is compared to the 0°–90°, 90°–180°, 180°–270°, and 270°–0° intervals of the indexing table. Using the known sum of the angles (360°) as the restraint for a least squares fit of the data, the angle of the block can be calculated. Note that there is no uncertainty in the restraint. The blocks between these extremes are more of a challenge.

The smaller angles are compared to subdivisions of a calibrated indexing table. For example, the 5° angle block is compared to each of the 5° subdivisions of a known 30° interval of a calibrated table. The calibrated value of this 30° interval is used as the restraint. Since we are not doing a 360° closure, this restraint does not have zero uncertainty. The 30° uncertainty is, however, apportioned to each of the six subdivisions, thereby reducing its importance in our final calculations. Thus the uncertainty from this calibration is not expected to be significantly higher than the full closure method.

To assess the reproducibility of the calibration we analyze the calibration history of our master blocks. From measurements caried out over a 30 year period, we find the standard devaition to be 0.073*″* (213 degrees of freedom). There is no apparent dependence on the size of the angle.

### 12.2 Long Term Reproducibility

Customer angle blocks are calibrated by comparison to the master angle blocks using two autocollimators set up so that each autocollimator is at null on a face of the master block [25]. The customer block is then put in the place of the master block and the two autocollimator readings are recorded. The scheme used is a drift eliminating design with two NIST master blocks used to provide both the restraint (sum of angles) and control (difference between angles) for the calibration. We estimate the reproducibility of the measurement from these control measurements.

Analysis of check standard data from calibrations performed over the last 10 years yields a standard deviation of 0.059*″* (380 degrees of freedom).

**Fig. 9:**
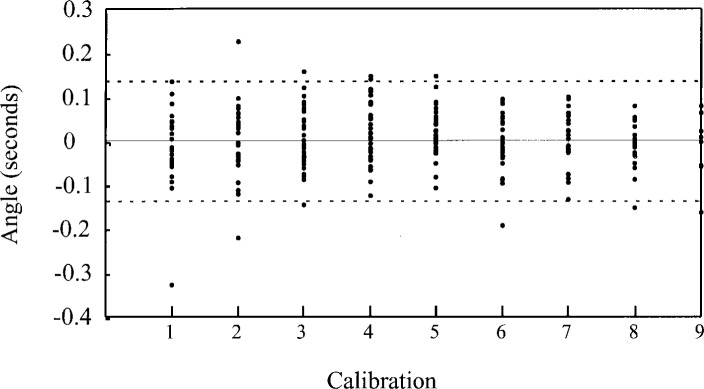
The variation of 16 gage blocks for 9 calibrations over 20 years. Each point is the measured deviation of a block from its historical mean calculated from the 9 calibrations.

Another check is to examine our customer historical data. Figure 9 shows a small part of that history: nine calibrations of one set of angle blocks over a 20 year period.

### 12.3 Thermal Expansion

Angle blocks are robust against angle changes caused by small homogeneous temperature changes. Tongs and gloves are used when handling the blocks to prevent temperature gradients that would cause angle errors. The blocks are measured in a small box and allowed to come to equilibrium before the data is taken, further reducing possible temperature effects. Any residual effects are sampled in the control history and are not listed separately.

### 12.4 Elastic Deformation

There is no mechanical contact.

### 12.5 Sensor Calibration

The uncertainty in the sensor (autocollimator) is the same as described in the earlier discussion of indexing tables.

### 12.6 Instrument Geometry

The only instrument geometry error arises if the angle block surface is not perpendicular to the autocollimator axis in the nonmeasuring direction. This error is a cosine error and is negligible in our setup.

### 12.7 Customer Artifact Geometry

Since the angle blocks are not exactly flat, it is possible that the surface area illuminated during the NIST calibration will not be the same area used by the customer. Since this is dependent on the customer’s equipment we do not include this source in our uncertainty budget. The possibility of errors arising from the use of the angle block in a manner different from our calibration is indicated in our calibration report.

### 12.8 Summary

Table 13 shows the uncertainty budget for angle block calibrations. The expanded uncertainty *U* (*k* = 2) is *U* = 0.18*″.*

## 13. Sieves

The Dimensional Metrology Group certifies wire mesh testing sieves to the current revision of ASTM Specification E-11[26]. We test the average wire diameter, average hole diameter, and the frame and skirt diameter. The frame and skirt diameters are checked with GO and NOGO gages, and therefore do not have an associated uncertainty.

Wire and hole diameters are measured with a calibrated optical projector. Hole diameters are measured indirectly; the pitch of the sieve is measured and the measured average wire diameter is subtracted to give the average hole size.

The uncertainty of the pitch (number of wires per centimeter) is very small. The sieve is mounted on an optical projector or traveling microscope. The sieve is moved until 100 wires have passed an index mark, and the pitch is calculated. For number 5 to number 50 sieves the number of wires is counted over a distance of 100 mm. The standard uncertainty of the measuring scale is less than 10 μm over any 100 mm of travel, giving a standard relative pitch uncertainty of 0.01 %. This is considerably smaller than the standard uncertainty of the wire diameter measurement and is ignored.

### 13.1 Master Artifact Calibration

Sieves are measured directly, so there are no master artifacts.

### 13.2 Long Term Reproducibility

We do not have check standards for sieve calibrations. We have, however, made multiple measurements on sieves using a number of different measuring methods.

For determining the pitch (average wire spacing) we have used different Moire scales, a traveling micrometer, and an optical projector to measure a single sieve. We find that the different methods all agree to within 0.5 μm or better for every sieve examined.

Measuring wire diameter optically is difficult because of diffraction effects at the edges of the wire. The diameter varies quite widely depending on the type of lighting (direction, coherence) and the quality of the optics. We have compared a number of different methods using back lighting, front lighting, diffuse and collimated light, and different optical systems. For these measurements both stage micrometers and calibrated wires have been used to calibrate the sensors. We find that these results agree within 2 μm. Having no clear theoretical reason to choose one method over the other, we take this spread as the uncertainty of optical methods. Taking the value of 2 μm as the half width of a rectangular distribution, we estimate the standard uncertainty to be 1 μm.

### 13.3 Thermal Expansion

The temperature control of our laboratory is adequate to make the uncertainty due to thermal effects negligible when compared to the tolerances required by the ASTM specification.

### 13.4 Elastic Deformation

There is no mechanical contact.

### 13.5 Sensor Calibration

The optical projector is calibrated with a precision stage micrometer. The stage micrometer has been calibrated at NIST and has a standard uncertainty of less than 0.03 μm. Since the optical comparator has a least count of only 1 μm, the stage micrometer length uncertainty is negligible. The uncertainty of the optical projector scale is taken as a rectangular distribution with half-width of 0.5 μm, giving an standard uncertainty of 0.29 um.

The correlation tests described earlier provide a practical test of the accuracy of the scale calibration.

### 13.6 Instrument Geometry

The major source of instrument uncertainty is the pitch error of the optical projector and traveling microscopes. Since both have large Abbe offsets the errors are as large as 20 μm. However, for fine sieves with tolerances of 3 μm to 10 μm, at least 300 wire spacings are measured to get the average pitch. For larger sieves, fewer wire spacings are measured but the tolerances are larger. In all cases the resulting error is far below the tolerance, and is ignored.

### 13.7 Customer Artifact Geometry

Customer sieves that have flatness problems are rejected as unmeasurable.

### 13.8 Summary

The major tests of sieves are the average wire and hole diameter. Since we calculate the hole diameter from the wire diameter and average wire spacing, the only non-negligible uncertainty is from the wire diameter measurement. Our experiments show that the variation between methods is much larger than the reproducibility of any one method. This variation between methods (two standard deviations, 95 % confidence) is taken as the expanded uncertainty *U* = 2 μm.

## Figures and Tables

**Table 1:** Uncertainty sources in NIST dimensional calibrations

1. Master Gage Calibration
2. Long Term Reproducibility
3. Thermal Expansion
a. Thermometer calibration
b. Coefficient of thermal expansion
c. Thermal gradients (internal, gage-gage, gage-scale)
4. Elastic Deformation
Probe contact deformation, compression of artifacts under their own weight
5. Scale Calibration
Uncertainty of artifact standards, linearity, fit routine
Scale thermal expansion, index of refraction correction
6. Instrument Geometry
Abbe offset and instrument geometry errors
Scale and gage alignment (cosine errors, obliquity, …)
Gage support geometry (anvil flatness, block flatness, …)
7. Artifact Effects
Flatness, parallelism, roundness, phase corrections on reflection

**Table 2:** Changes in environmental conditions that produce the indicated fractional changes in the wavelength of light

	Fractional change in wavelength		
Environmental parameter	1×10^−6^	1×10^−7^	1×10^−8^
Temperature	1 °C	0.1 °C	0.01 °C
Pressure	400 Pa	40 Pa	4 Pa
Water vapor pressure at 20 °C	2339 Pa	280 Pa	28 Pa
Relative humidity	100 %, saturated	12 %	1.2 %
CO_2_ content (volume fraction in air)	0.006 9	0.000 69	0.000 069

**Table 3:** Uncertainty budget for NIST master gage blocks

	Source of uncertainty	Standard uncertainty (*k* = 1)
1.	Master gage calibration	N/A
2.	Long term reproducibility	0.009 μm+0.08×10^−6^ *L*
3.	Thermometer calibration	N/A
4.	CTE	0.005×10^−6^ *L*
5.	Thermal gradients	0.030×10^−6^ *L* up to *L*=0.1 m
6.	Elastic deformation	Negligible
7.	Scale calibration	0.003×10^−6^ *L*
8.	Instrument geometry	Negligible
9.	Artifact geometry—phase correction	0.006 μm

**Table 4:** Uncertainty budget for NIST customer gage blocks measured by interferometry

	Source of uncertainty	Standard uncertainty (*k* = 1)
1.	Master gage calibration	N/A
2.	Long term reproducibility	0.022 μm+0.2×10^−6^ *L*
3.	Thermometer calibration	N/A
4.	CTE	0.060×10^−6^ *L*
5.	Thermal gradients	0.030×10^−6^ *L* up to *L*=0.1 m
6.	Elastic deformation	Negligible
7.	Scale calibration	0.003×10^−6^ *L*
8.	Instrument geometry	Negligible
9.	Artifact geometry—phase correction	0.006 μm
10.	Artifact geometry—gage point position	0.003 μm

**Table 5:** Standard uncertainty for length of NIST master gage blocks

Type of block	Standard uncertainty
Thin (<1 mm)	0.008 μm
Intermediate (1 mm to 100 mm)	0.004 μm+0.12×10^−6^ *L*
Long (>100 mm)	0.020 μm+0.03×10^−6^ *L*

**Table 6:** Uncertainty budget for NIST customer gage blocks measured by mechanical comparison

Source of uncertainty		Standard uncertainty (*k* = 1)		

		Thins (<1 mm)	1 mm to 100 mm	over 100 mm
1.	Master gage cal.	0.012 μm	0.012 μm+0.08×10^−6^ *L*	0.012 μm+0.08×10^−6^ *L*
2.	Reproducibility	0.008 μm	0.004 μm+0.12×10^−6^ *L*	0.020 μm+0.03×10^−6^ *L*
3a.	Thermometer cal.	Negligible	negligible	negligible
3b.	CTE	0.08×10^−6^ *L*	0.08×10^−6^ *L*	0.04×10^−6^ *L*
3c.	Thermal Gradients	0.17×10^−6^ *L*	0.17×10^−6^ *L*	0.07×10^−6^ *L*
4.	Elastic Deformation	0.002 μm	0.002 μm	0.002 μm
5.	Scale Calibration	0.002 μm	0.002 μm	0.020 μm
6.	Instrument Geometry	0.010 μm	0.002 μm	0.002 μm
7.	Artifact Geometry	0.008 μm	0.008 μm	0.008 μm

**Table 7:** Uncertainty budget for NIST customer gage wires and cylinders measured by mechanical comparison

	Source of uncertainty	Standard uncertainty (*k* = 1)
1.	Master gage calibration	0.065 μm
2.	Long term reproducibility	0.025 μm
3a.	Thermometer calibration	Negligible
3b.	CTE	0.01×10^−6^ *L*
3c.	Thermal gradients	0.017×10^−6^ *L*
4.	Elastic deformation	0.013 μm
5.	Scale calibration	N/A
6.	Instrument geometry	N/A
7.	Artifact geometry	0.008 μm

**Table 8:** Uncertainty budget for NIST customer gage blocks measured by mechanical comparison

	Source of uncertainty	Standard uncertainty (*k* = 1)
1.	Master gage calibration	0.038 μm+0.2×10^−6^ *L*
2.	Long term reproducibility	0.025 μm
3a.	Thermometer calibration	N/A
3b.	CTE	0.12×10^−6^ *L*
3c.	Thermal gradients	N/A
4.	Elastic deformation	0.005 μm
5.	Scale calibration	0.003 μm
6.	Instrument geometry	0.010 μm
7.	Artifact geometry	Negligible

**Table 9:** Uncertainty budget for NIST customer gage balls measured by mechanical comparison

Source of uncertainty		Standard uncertainty (*k* = 1)	

		Uncertainty (general)	Uncertainty (30 mm ball)
1.	Master gage cal.	0.040 μm	0.040 μm
2.	Reproducibility	0.035 μm	0.035 μm
3a.	Thermometer cal.	0.08×10^−6^ *L*	0.003 μm
3b.	CTE	0.20×10^−6^ *L*	0.006 μm
3c.	Thermal Gradients	0.17×10^−6^ *L*	0.005 μm
4.	Elastic Deformation	0.010 μm	0.010 μm
5.	Scale Calibration	0.003 μm	0.003 μm
6.	Instrument Geometry	Negligible	Negligible
7.	Artifact Geometry	As needed	As needed

**Table 10:** Uncertainty budget for NIST customer roundness standards

	Source of uncertainty	Standard uncertainty (*k* = 1)
1.	Master gage calibration	N/A
2.	Long term reproducibility	0.008 μm
3a.	Thermometer calibration	N/A
3b.	CTE	N/A
3c.	Thermal gradients	N/A
4.	Elastic deformation	N/A
5.	Scale calibration	0.002 μm
6.	Instrument geometry	N/A
7.	Artifact geometry	N/A

**Table 11:** Uncertainty budget for NIST customer optical flats

	Source of uncertainty	Standard uncertainty (*k* = 1)
1.	Master gage calibration	0.0026 μm
2.	Long term reproducibility	0.0030 μm
3a.	Thermometer calibration	N/A
3b.	CTE	N/A
3c.	Thermal gradients	Negligible
4.	Elastic deformation	Negligible
5.	Scale calibration	Negligible
6.	Instrument geometry	Negligible
7.	Artifact geometry	N/A

**Table 12:** Uncertainty budget for NIST customer indexing tables

	Source of uncertainty	Standard uncertainty (*k* = 1)
1.	Master gage calibration	N/A
2.	Long term reproducibility	0.07*″*
3a.	Thermometer calibration	N/A
3b.	CTE	N/A
3c.	Thermal gradients	N/A
4.	Elastic deformation	N/A
5.	Scale calibration	0.01*″*
6.	Instrument geometry	N/A
7.	Artifact geometry	N/A

**Table 13:** Uncertainty budget for NIST customer angle blocks

	Source of uncertainty	Standard uncertainty (*k* = 1)
1.	Master gage calibration	0.075*″*
2.	Long term reproducibility	0.060*″*
3a.	Thermometer calibration	N/A
3b.	CTE	N/A
3c.	Thermal gradients	N/A
4.	Elastic deformation	N/A
5.	Scale calibration	0.010*″*
6.	Instrument geometry	N/A
7.	Artifact geometry	N/A
